# Supplementary material for: The health costs of losing political representation: Evidence from U.S. Presidential Elections
Source: PLoS One. 2025 Oct 31;20(10):e0334507. doi: 10.1371/journal.pone.0334507 (PMC12578145; doi:10.1371/journal.pone.0334507)
Supplement: S1 Text — (PDF) [file pone.0334507.s001.pdf]

Online Appendix

This Appendix is for Online Publication and provides further details on the data and the results of the paper “The Health Costs of Losing Political Representation: Evidence from U.S. Presidential Elections”.

S1 Figures

S1.1 Further analysis of the parallel trend assumption.

Figure S1 shows the results of a sensitivity test following the approach proposed by [66]. This procedure involves an examination of the extent to which our main results remain robust in the face of potential nonlinearities with varying magnitudes in the counterfactual trend. More specifically, we calculate 95 % confidence intervals for our main estimators under varying assumptions of the value M, the upper limit for the change between two consecutive time periods in the slope of the underlying linear trend. A value of M equal to 0 on the x-axis corresponds with allowing for linear violations of parallel trends. Larger values of M allow for larger deviations from linearity. We show that the fixed length confidence intervals are similar to those from our baseline estimators when allowing for violations of parallel trends that are approximately linear. Our results are also robust to allow for larger degrees of possible non-linearity in the violation of parallel trends. The breakdown value for a significant effect is indeed equal to a value of M of around 0.6 for the event study related to the Obama Presidential election and 0.4 for the event study related to the Trump Presidential election.

Figure S1: Sensitivity test

Notes: Figure S1 provides the results of a formal sensitivity analysis that relates the magnitude of violations of parallel trends to the robustness of treatment estimates in post-treatment periods [66]. It shows 95 % confidence intervals for our main estimators under varying assumptions of the value M.

S1.2 Black Mortality and Obama.

Figure S2(a) and Figure S2(b) show respectively the time-series trends of average black and other races mortality rates and the yearly treatment effects estimated using a dynamic version of Equation (8). Together, the Figure supports the hypothesis that black mortality rates decreased after the election of Obama.

Figure S2: Obama and black mortality rates

Notes: Figure S2(a) shows the time-series trends of black and other races mortality rates after the election of Obama. Figure S2(b) shows the yearly treatment using a dynamic version of Equation (8) with county, year times state and race times state fixed effects. The dependent variable is Mortality. Standard errors are double clustered at the county and year level. See section S2 for a detailed description of every variable.

S1.3 Added Variable Plot.

We want to test whether our results are not driven by outliers. We analyze this issue graphically and report the added variable plots for Equation (3) in Figure S3.

The figures show the relationship between the age-adjusted mortality rates and the vote shares for the Republican and Democrat parties after the 2008 and 2016 Presidential elections respectively while simultaneously accounting for the influence of all the other control variables and fixed effects. We do not find any evidence that our main findings are driven by a subset of observations.

Figure S3: Added variable plots

Notes: Figure S3 shows binned added variable plots for the age-adjusted mortality rates and the vote shares for the Republican and Democrat parties after the 2008 and 2016 Presidential elections, respectively.

S1.4 Anticipation in Obama election?

In Figure 1, there is a noticeable dip in mortality trends for both Democratic and Republican counties between 2006 and 2007, before the election of Obama. This could reflect a broader national improvement in health outcomes affecting all counties (for example, due to aggregate policy changes or demographic shifts). However, it is also important to notice a more substantial decrease in the mortality rate in Democratic counties. In order to test whether this difference is statistically significant and whether it leads to a violation of the parallel trends assumption, we re-estimated Equation (3) using 2006 as the reference year. The results are reported in Figure (S4).

Figure S4: Alternative reference year

Notes: Figure S4 shows the yearly treatment effects and the 95 % confidence intervals from Equation (3) with county and year times states fixed effects. The dependent variable is *Mortality*, and the event is the turnover Presidential elections of 2008. We use as a treatment an indicator variable equal to one if the share of voters during the turnover Presidential election is greater than the median value, and zero otherwise. Standard errors are double clustered at the county and year level. See section S2 for a detailed description of every variable.

S1.5 Internal and external causes of death.

In an additional exercise, we analyze the dynamic effects of internal and external causes of death—the two primary categories defining mortality. The results are presented in Figures (S5) and (S6). We still find that the assumption of parallel trends is likely to hold. Additionally, our analysis shows that internal causes of death are the primary determinants of our results, consistent with the hypothesis that sentiments drive our findings. On the other hand, the coefficients related to external causes of death are all close to zero and mostly not statistically significant at the conventional 90% levels.

Figure S5: Internal mortality rates

Notes: Figure 2 shows the yearly treatment effects and the 95 % confidence intervals from Equation (3) with county and year times states fixed effects. The dependent variable is *Internal Mortality Rates* and the events are the two turnover Presidential elections of 2008 and 2016. We use as a treatment an indicator variable equal to one if the share of voters during the turnover Presidential election is greater than the median value, and zero otherwise. Standard errors are double clustered at the county and year level. See section S2 for a detailed description of every variable.

Figure S6: External morality rates

Notes: Figure 2 shows the yearly treatment effects and the 95 % confidence intervals from Equation (3) with county and year times states fixed effects. The dependent variable is *External Mortality Rates* and the events are the two turnover Presidential elections of 2008 and 2016. We use as a treatment an indicator variable equal to one if the share of voters during the turnover Presidential election is greater than the median value, and zero otherwise. Standard errors are double clustered at the county and year level. See section S2 for a detailed description of every variable.

S1.6 Alternative treatment variable.

We follow [18] and consider an alternative specification in which we propose as a treatment an indicator variable equal to one if the share of voters during the turnover Presidential election is greater than the median value, and zero otherwise. As reported in Figure S7, we consistently find a negative effect on mortality after turnover Presidential elections and also show that the standard parallel trend assumption is likely to hold in this setting considering the absence of significant pre-event treatment coefficients.

Figure S7: Alternative treatment

Notes: Figure 2 shows the yearly treatment effects and the 95 % confidence intervals from Equation (3) with county and year times states fixed effects. The dependent variable is *Mortality* and the events are the two turnover Presidential elections of 2008 and 2016. We use as a treatment an indicator variable equal to one if the share of voters during the turnover Presidential election is greater than the median value, and zero otherwise. Standard errors are double clustered at the county and year level. See section S2 for a detailed description of every variable.

S2 Tables

S2.1 Age-Adjusted Mortality Rates

We use proprietary information on death certificates for United States residents from the U.S. Centers for Disease Control (CDC). Between 2000 and 2019, the number of people that has been reported deceased in our database is equal to 51’125’902.

We count the number of individuals who died by county of residence and year. We use this information and population estimates from the National Cancer Institute’s Surveillance, Epidemiology End Results (SEER) Program to compute age-adjusted mortality rates (per 100’000 population), a weighted average of the crude death rates across age categories within a county, where the shares of the overall US population in each age category are used as weights.

The age categories we consider in our analysis are less than 1 year, 1 - 4 years, 5 - 14 years, 15-24 years, 25-34 years, ... and older than 85 years. In order to compute the weights, we consider U.S. population shares in the year 2000. We report the weights that we use in Table S1.

Table S1: Population Weights

**Age Category**	Weights
0	0.0136657
1-4 years	0.0543033
5-14 years	0.1456663
15-24 years	0.1396771
25-34 years	0.1410695
35-44 years	0.160081
45-54 years	0.1346706
55-64 years	0.086578
65-74 years	0.0651537
75-84 years	0.0440284
85 years	0.0151064

Notes: The table shows population weights per age category that we use to build our age adjusted mortality rates.

S2.2 Description of the variables.

Table S2 contains detailed information on the variables that we use in the empirical analysis, their definition, and their sources.

Table S2: Variable description

Variable name	Description	Source
**Panel A: County Variables**		
Mortality	Age-adjusted mortality rate per 100,000 county population. Rates are computed as a weighted average of the crude death rates across age categories within a county, where the shares of the overall U.S. population in each age category are used as weights	CDC
Democrats	The share of votes for the Democrat candidate during the 2016 Presidential election	MIT
Republicans	The share of votes for the Republican candidate during the 2008 Presidential election	MIT
Electoral Loss	Dummy variable equal to 1 if the county political preferences (using as threshold the median value of the Democrat variable distribution) is the same of the party of the President	MIT
Income	The natural logarithm of the county income per capita	BLS
Population	The natural logarithm of the county population	BLS
Unemployment	The county unemployment rate	BLS
Employment	The natural logarithm of the number of employees	CBP
Establishments	The natural logarithm of the number of establishments	CBP
Wages	The natural logarithm of the total annual payroll ($1,000)	CBP
Transfers	The natural logarithm of income payments to persons for which no current services are performed and net insurance settlements. It is the sum of government social benefits and net current transfer receipts from business	BLS
HPI	A weighted, repeat-sales index, that measures average price changes in repeat sales or refinancings on the same properties	FHFA
Polarization	The absolute difference between a county's political preferences for the Democratic party and the political preferences of the neighboring counties for the same party	MIT
Share Independent	The share of the votes for other parties in the Presidential elections	MIT
Share No Voters	The share of people in the county that did not vote in the Presidential election	MIT
Membership	The total number of membership associations divided by 10'000 population. We identify membership associations using the NAICS code (813410, 713950, 713910, 713940, 711211, 813110, 813940, 813930, 813910, and 813920)	NETS

Notes: This table shows a detailed description of each variable and its source.

Table S2: Variable description cont'd

**Panel B: BRFSS**		
General Health	A self-rated measure of general health measured considering the following question: *"Would you say that in general your health is excellent, very good, good, fair, poor?"*. We assigned a maximum value of 4 to "Excellent" and a minimum value of 0 to "Poor"	BRFSS
Mental Health	A self-rated measure of mental health measured considering the following question: *"Now thinking about your mental health, which includes stress, depression, and problems with emotions, for how many days during the past 30 days was your mental health not good?"*. The variable spans from a minimum of 0 to a maximum value of 30	BRFSS
Age	The age of the individuals replying to the survey	BRFSS
Income	Eight categorical variables for the category of the income of the individual	BRFSS
Female	Dummy variable equal to 1 if the respondent declares to be a female	BRFSS
Marital Status	Dummy variable equal to 1 if the respondent declares to be married	BRFSS
**Panel C: GSS**		
General Health	A self-rated measure of general health measured considering the following question: *"Would you say that in general your health is excellent, very good, good, fair, poor?"*. We assigned a maximum value of 4 to "Excellent" and a minimum value of 0 to "Poor"	GSS
Democrat	A self-rated measure of political identity measured considering the following question: *"Generally speaking, do you usually think of yourself as a Republican, Democrat, Independent, or what??"*. We assigned a value of 1 if the individual replied "Strong Democrat" and 0 otherwise	GSS
Republican President	A dummy variable equal to one if the president is a Republican	Wikipedia
Age	The age of the individuals replying to the survey	GSS
Income	Twelve categorical variables for the category of the income of the individual	GSS
Female	Dummy variable equal to 1 if the respondent declares to be a female	GSS
Marital Status	Dummy variable equal to 1 if the respondent declares to be married	GSS

Notes: This table shows a detailed description of each variable and its source.

S2.3 Mental health robustness.

Since the BRFSS survey is not representative at the county level but rather at the state level, we show in Table S3 that our results hold when we measure our treatment variable (the share of Republican voters) at the state level.

Table S3: Mental Health

Variables	(1)<br>Mental Health > 0	(2)<br>Mental Health > 0	(3)<br>Log(Mental Health)
Post × Republicans	0.0377***	0.0413***	0.0709**
	(0.0113)	(0.0138)	(0.0274)
Age		0.0044***	0.0192***
		(0.0002)	(0.0005)
Age squared		-0.0001***	-0.0003***
		(0.0000)	(0.0000)
Married		-0.0397***	-0.0956***
		(0.0010)	(0.0025)
Female		0.0959***	0.1801***
		(0.0010)	(0.0022)
Income = 2		-0.0438***	-0.1725***
		(0.0020)	(0.0058)
Income = 3		-0.0831***	-0.3084***
		(0.0020)	(0.0067)
Income = 4		-0.1101***	-0.4081***
		(0.0023)	(0.0075)
Income = 5		-0.1419***	-0.5192***
		(0.0023)	(0.0072)
Income = 6		-0.1611***	-0.5939***
		(0.0025)	(0.0078)
Income = 7		-0.1806***	-0.6670***
		(0.0028)	(0.0085)
Income = 8		-0.2173***	-0.7681***
		(0.0030)	(0.0091)
County FE	Yes	Yes	Yes
Time FE	Yes	Yes	Yes
Observations	2,954,844	2,954,844	2,954,844
Adjusted R-squared	0.00462	0.0700	0.0785

Notes: This table shows regression results for Equation (5). *Mental Health* is the dependent variable and is a dummy variable equal to 1 if (or the natural logarithm of) the number of days in the last month the individual reported his/her mental health was not good is at least one. Mental health issues include stress, depression, and problems with emotions. Standard errors are double clustered at the county and time level. ***, **, and * denote significance at 1, 5, and 10 percent level respectively. See section S1 of the online appendix for a detailed description of every variable.

S2.4 A comparison of losing and winning counties.

Table S4 shows the summary statistics of losing and winning counties. It also shows the normalized difference in the last column, which indicates similarity between both groups if this value is within the range of ±0.25 [81]. Only three out of eight variables are outside this range.

Table S4: Normalized Differences

	Winning Counties		Losing Counties		
	Mean	SD	Mean	SD	ND
Income(log)	10.46	0.27	10.34	0.31	0.29
Population(log)	10.24	1.50	10.10	1.40	0.06
Unemployment(log)	6.94	2.89	5.12	2.21	0.50
Establishment(log)	6.41	1.52	6.30	1.41	0.05
Employment(log)	8.75	1.98	8.65	1.76	0.03
Wages(log)	12.22	2.13	12.01	1.97	0.07
HPI(log)	4.89	0.17	4.86	0.20	0.13
Trasnfers(log)	8.95	0.27	8.76	0.39	0.39

Notes: The table shows descriptive statistics for the county characteristics. We separate the sample into losing and winning counties. The last column provides normalized differences [81]. See section S1 of the online appendix for a detailed description of every variable.

S2.5 Obama and black mortality rates.

We exploit a unique characteristic of the Obama Presidential election; most of the black voters (95 % of the black population) cast their ballot for Democrat Barack Obama (Pew Research Center, 2009). Exploiting this peculiarity, we build a new database at county-race-year level and compare black individuals' mortality rates with the mortality rates of other races (White, Hispanic, and Asiatic), living in the *same county* during the same period. We show estimation results from Equation 8 in Table S5.

Table S5: Black Individuals

Variables	(1) Mortality	(2) Mortality	(3) Mortality
Post × Black	-35.1221**	-34.4535**	-35.9724**
	(10.6157)	(11.1740)	(11.5380)
County FE	Yes	Yes	Yes
Year FE	Yes	Yes	Yes
Race FE	Yes	Yes	Yes
Race-Year FE	No	Yes	Yes
State-Year FE	No	Yes	Yes
Post × County Controls	No	No	Yes
Observations	112,736	112,736	103,369
Adjusted R-squared	0.0978	0.151	0.150

Notes: This table shows regression results for equation (8). Mortality is the dependent variable and is the race-age-adjusted mortality rate. Standard errors are double clustered at the county and year level. ***, **, and * denote significance at 1, 5, and 10 percent level respectively. See section S1 of the online appendix for a detailed description of every variable.

S2.6 Outliers.

We show that our results do not change if we winsorize all the variables at the first and the last percentiles. We report the results in Table S6. Our coefficients of interest are still statistically significant. In terms of magnitude, they are slightly smaller but within one standard deviation of our baseline results.

Table S6: Outliers

Variables	(1) Mortality	(2) Mortality	(3) Mortality
Panel A: Obama and Mortality			
Post × Republicans	41.8002***	45.9572***	41.2698**
	(9.0094)	(12.0122)	(13.6534)
County FE	Yes	Yes	Yes
Year FE	Yes	Yes	Yes
State-Year FE	No	Yes	Yes
Post × Controls	No	No	Yes
Observations	28,017	28,017	27,540
Adjusted R-squared	0.730	0.735	0.733
Panel B: Trump and Mortality			
Post × Democrats	17.9666*	21.4956*	23.9830**
	(9.1956)	(9.8313)	(10.1013)
County FE	Yes	Yes	Yes
Year FE	Yes	Yes	Yes
State-Year FE	No	Yes	Yes
Fully interacted controls	No	No	Yes
Observations	24,912	24,904	24,480
Adjusted R-squared	0.765	0.766	0.765

Notes: This table shows regression results for equation (4). *Mortality* is the dependent variable and is the age-adjusted mortality rate of the county. We winsorize all the variables at the first and last percentiles. Standard errors are double-clustered at the county level. ***, **, and * denote significance at 1, 5, and 10 percent level respectively. See section S1 of the online appendix for a detailed description of every variable.

S2.7 Standard errors.

We show that our results do not change if we cluster our coefficients of interest at different levels. More specifically, we cluster standard errors respectively at the county, at the county and year level, at the state level, and at the state and year level and we report estimation results in Table S7.

Table S7: Clustering

Variables	(1) Mortality	(2) Mortality	(3) Mortality	(4) Mortality
Panel A: Obama and Mortality				
Post × Republicans	44.6786***	44.6786**	44.6786**	44.6786**
	(14.8596)	(15.2342)	(19.7121)	(18.4464)
County FE	Yes	Yes	Yes	Yes
Year FE	Yes	Yes	Yes	Yes
State-Year FE	Yes	Yes	Yes	Yes
Post × Controls	Yes	No	Yes	Yes
Cluster	County	County and Year	State	State and Year
Observations	27,540	27,540	27,540	27,540
Adjusted R-squared	0.703	0.703	0.703	0.703
Panel B: Trump and Mortality				
Post × Democrats	30.9178**	30.9178**	30.9178**	30.9178*
	(14.2460)	(12.3927)	(15.3085)	(13.1430)
County FE	Yes	Yes	Yes	Yes
Year FE	Yes	Yes	Yes	Yes
State-Year FE	Yes	Yes	Yes	Yes
Post × Controls	Yes	No	Yes	Yes
Cluster	County	County and Year	State	State and Year
Observations	24,480	24,480	24,480	24,480
Adjusted R-squared	0.747	0.747	0.747	0.747

Notes: This table shows regression results for equation (4) when we cluster the standard errors at different level. *Mortality* is the dependent variable and is the age-adjusted mortality rate of the county. ***, **, and * denote significance at 1, 5, and 10 percent level respectively. See section S1 of the online appendix for a detailed description of every variable.

S2.8 The election of Obama and the Great Recession.

We include in our regression a measure of the severity of the Great Recession. We follow [77] and quantify the severity of the Great Recession using unemployment changes between 2007 and 2009 in each county. We report the estimation results in Table S8 and show that our findings are not affected.

Table S8: The Great Recession

Variables	(1) Mortality	(2) Mortality	(3) Mortality
Post $\times$ Republicans	43.0299***	49.3701***	44.9463**
	(11.5373)	(13.8752)	(15.5446)
Post $\times$ Severity	-17.9114**	-8.9031	-2.5961
	(5.5928)	(6.4375)	(7.0448)
County FE	Yes	Yes	Yes
Year FE	Yes	Yes	Yes
State-Year FE	No	Yes	Yes
County controls	No	No	Yes
Observations	28,008	28,008	27,540
Adjusted R-squared	0.700	0.705	0.703

Notes: This table shows regression results for equation (4) and we control for the severity of the Great Recession. *Mortality* is the dependent variable and is the age-adjusted mortality rate of the county. Standard errors are double clustered at the county and year level. ***, **, and * denote significance at 1, 5, and 10 percent level respectively. See section S1 of the online appendix for a detailed description of every variable.

S2.9 Placebo tests.

We analyze the effect of turnover elections on the health of non-registered voters and independents. To do so, we consider two variables: i. the share of votes for the independent party and ii. the share of people in the county who did not vote during the election. We next estimate again Equation (3). Furthermore, we also consider a placebo test based on the Presidential election of 2012 (spanning period 2008-2015). Since Obama won the election again, there has not been any political turnover. We also standardize these variables to make them easy to compare with each other. We report the results in Table S9.

Table S9: Placebo tests

Variables	(1) Mortality	(2) Mortality	(3) Mortality	(4) Mortality
Panel A: Obama				
Post × Share Republicans (Baseline)	6.169***			
	(2.052)			
Post × Share No Voters		5.222		
		(3.248)		
Post × Share Independent			1.358	
			(3.115)	
Post × Share Republicans (Placebo)				3.013
				(1.969)
County FE	Yes	Yes	Yes	Yes
Year FE	Yes	Yes	Yes	Yes
State-Year FE	Yes	Yes	Yes	Yes
County controls	Yes	Yes	Yes	Yes
Observations	27,540	27,540	27,540	22,856
Adjusted R-squared	0.703	0.703	0.703	0.768
Panel B: Trump				
Post × Share Democrats (Baseline)	4.706**			
	(1.886)			
Post × Share Did not Vote		1.401		
		(1.872)		
Post × Share Independent			0.631	
			(3.821)	
Post × Share Democrats (Placebo)				-2.954
				(1.983)
County FE	Yes	Yes	Yes	Yes
Year FE	Yes	Yes	Yes	Yes
State-Year FE	Yes	Yes	Yes	Yes
County controls	Yes	Yes	Yes	Yes
Observations	24,480	24,480	24,480	22,856
Adjusted R-squared	0.747	0.747	0.747	0.768

Notes: This table shows regression results for Equation (4) when we consider alternative measures of county political preferences and a placebo test base on the Presidential election of 2012. *Mortality* is the dependent variable and is the age-adjusted mortality rate of the county. ***, **, and * denote significance at 1, 5, and 10 percent level respectively. See section S1 of the online appendix for a detailed description of every variable.

S2.10 General health.

We show that our results hold when we consider a self-reported measure of general health status from the BRDSS. We estimate Equation (5) using as outcome variable of interest a measure of general health status that we obtain considering the following question: *"Would you say that in general your health is excellent, very good, good, fair, poor?"*. We assigned a maximum value of 4 to "Excellent" and a minimum value of 0 to "Poor". In line with our main results, we find that general health gets worst in Republican counties after the Obama election.

Table S10: General Health

Variables	(1)<br>General Health	(2)<br>General Health
Post × Republicans	-0.0328*	-0.0432***
	(0.0167)	(0.0129)
Age		-0.0287***
		(0.0006)
Age squared		0.0002***
		(0.0000)
Married		-0.0393***
		(0.0024)
Female		0.0865***
		(0.0023)
Income = 2		0.1471***
		(0.0059)
Income = 3		0.3496***
		(0.0077)
Income = 4		0.5328***
		(0.0091)
Income = 5		0.7268***
		(0.0084)
Income = 6		0.9118***
		(0.0092)
Income = 7		1.0800***
		(0.0097)
Income = 8		1.3117***
		(0.0097)
County FE	Yes	Yes
Time FE	Yes	Yes
Observations	2,929,186	2,929,186
Adjusted R-squared	0.0294	0.184

Notes: This table shows regression results for Equation (5). *General Health* is the dependent variable. It is a measure of general health status that we obtain considering the following question: *"Would you say that in general your health is excellent, very good, good, fair, poor?"*. We assigned a maximum value of 4 to "Excellent" and a minimum value of 0 to "Poor" . Standard errors are double clustered at the county and time level. ***, **, and * denote significance at 1, 5, and 10 percent level respectively. See section S1 of the online appendix for a detailed description of every variable.

S2.11 A long survey database.

Our results are based on two turnover Presidential elections that take place between 2000 and 2019. To provide external validity to our results, we show that our conclusions hold when we consider a long survey database starting in 1973. We report estimation results from Equation (9) in Table S11.

Table S11: The GSS

Variables	(1) Health	(2) Health	(3) Health
Democrat	-0.0964***	-0.0832***	0.0015
	(0.0162)	(0.0162)	(0.0154)
Republican President × Democrat	-0.0650***	-0.0705***	-0.0353*
	(0.0225)	(0.0225)	(0.0211)
Republican President	0.0337***		
	(0.0086)		
Female			0.0047
			(0.0076)
Age			-0.0232***
			(0.0013)
Age Squared			0.0001***
			(0.0000)
Married			0.0364***
			(0.0083)
Income = 2			-0.0897**
			(0.0453)
Income = 3			-0.1412***
			(0.0461)
Income = 4			-0.0301
			(0.0472)
Income = 5			0.0141
			(0.0460)
Income = 6			0.0221
			(0.0456)
Income = 7			0.1047**
			(0.0448)
Income = 8			0.1137***
			(0.0411)
Income = 9			0.2158***
			(0.0375)
Income = 10			0.2938***
			(0.0379)
Income = 11			0.3665***
			(0.0379)
Income = 12			0.5874***
			(0.0363)
Constant	2.0357***		
	(0.0060)		
Region FE	No	Yes	Yes
Year FE	No	Yes	Yes
Other controls	No	No	Yes
Observations	43,943	43,943	43,943
R-squared	0.0038	0.0133	0.1198

Notes: This table shows regression results for Equation (9). *General Health* is the dependent variable. It is a measure of general health status that we obtain considering the following question: *"Would you say that in general your health is excellent, very good, good, fair, poor?"*. We assigned a maximum value of 4 to "Excellent" and a minimum value of 0 to "Poor". Standard errors are adjusted for heteroskedasticity. ***, **, and * denote significance at 1, 5, and 10 percent level respectively. See section S1 of the online appendix for a detailed description of every variable.

S2.12 Additional controls.

We demonstrate in Table S12 the robustness of our main results by incorporating and interacting with Post the following additional control variables into our main specification: a) the median age in the county, b) the percentage with a BA or higher, c) the percentage with health insurance in each county, and d) a dummy variable equal to one for minority counties.

Table S12: Additional control variables

**Variables**	(1)<br>Mortality	(2)<br>Mortality
Post × Republicans	39.184**<br>(14.901)	
Post × Democrats		32.880**<br>(12.199)
County FE	Yes	Yes
Year FE	Yes	Yes
State-Year FE	Yes	Yes
Interacted controls + Addtional controls	Yes	Yes
Observations	27,531	24,472
Adjusted R-squared	0.703	0.747

Notes: This table shows regression results for Equation (4). *Mortality* is the dependent variable and is the age-adjusted mortality rate in the county. We additional control for: a) the median age in the county, b) the percentage with a BA or higher, c) the percentage with health insurance in each county, and d) a dummy variable equal to one if the county is a minority county. Standard errors are double clustered at the county and year level. ***, **, and * denote significance at 1, 5, and 10 percent level respectively. See section S1 of the online appendix for a detailed description of every variable.
