# Supplementary figures and images for: The health costs of losing political representation: Evidence from U.S. Presidential Elections
Source: PLoS One. 2025 Oct 31;20(10):e0334507. doi: 10.1371/journal.pone.0334507 (PMC12578145; doi:10.1371/journal.pone.0334507)

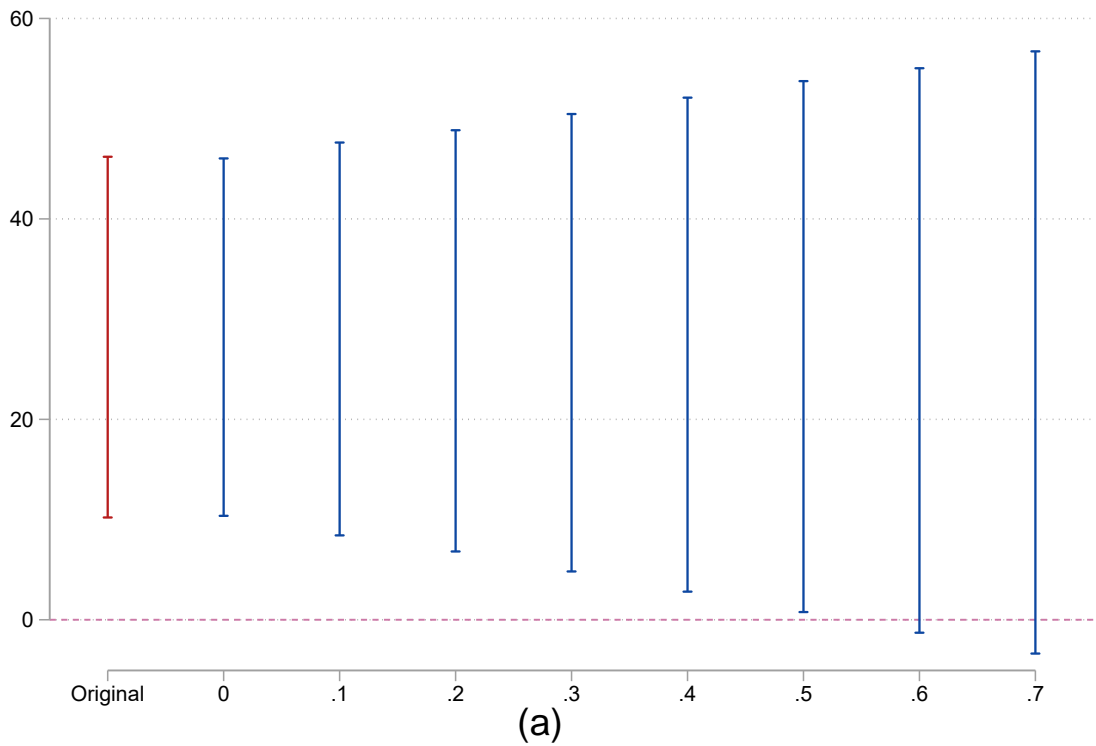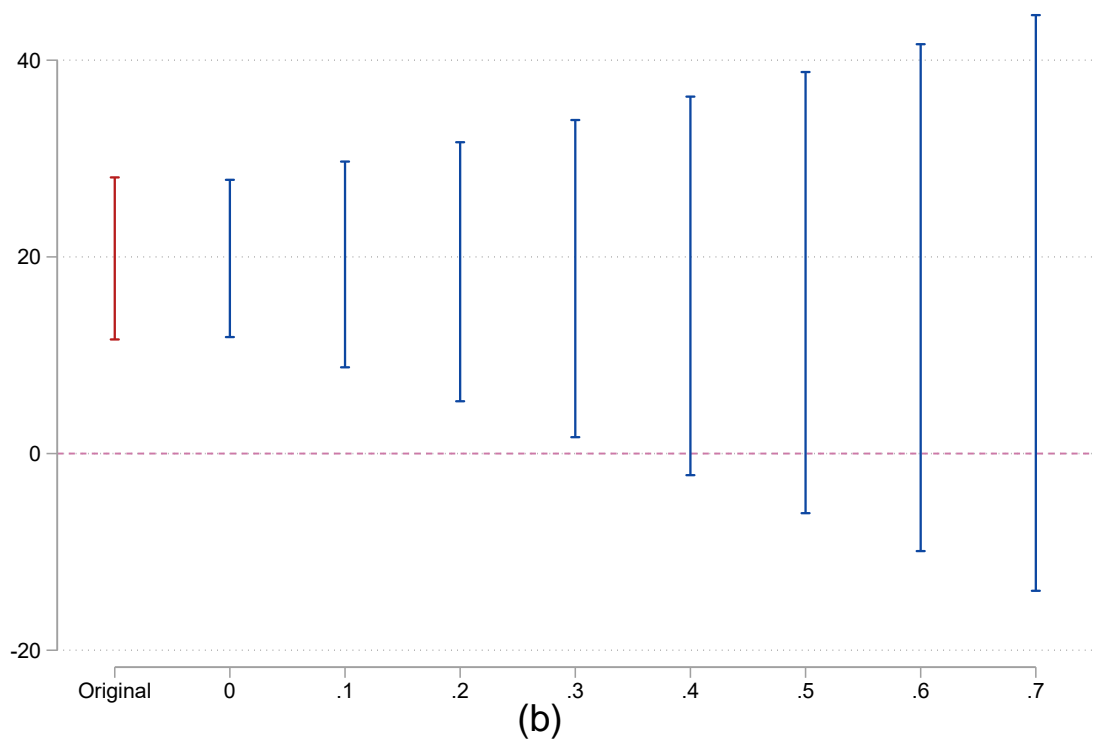

Supplement: S1 Fig — (PDF) [file pone.0334507.s002.pdf]

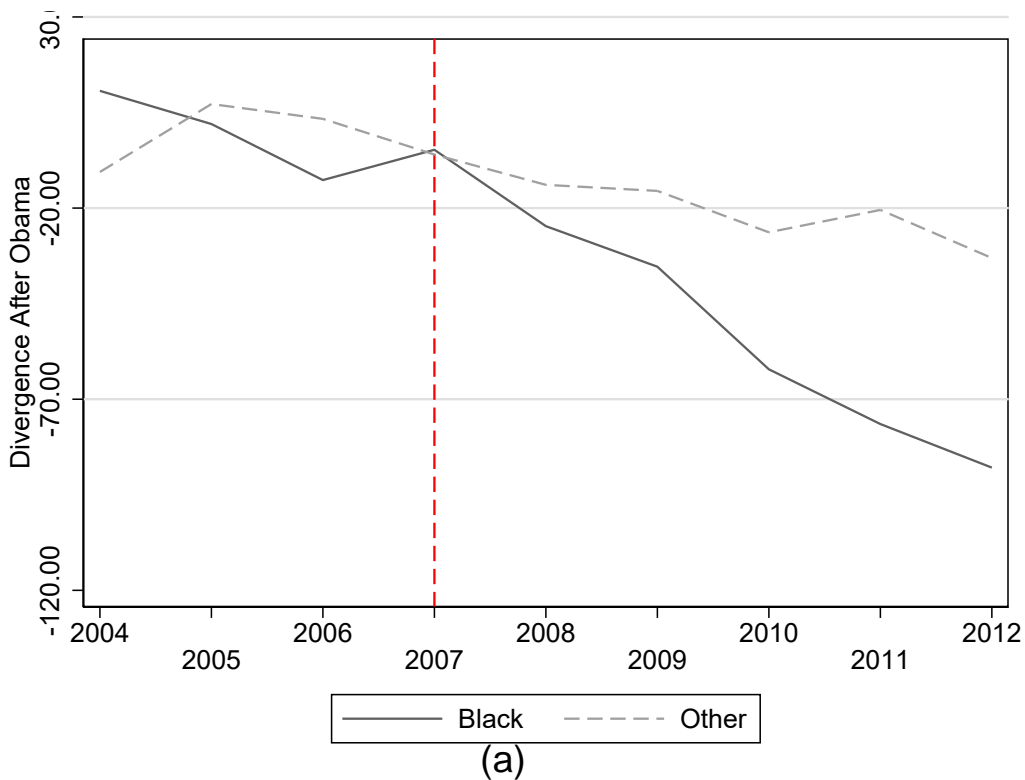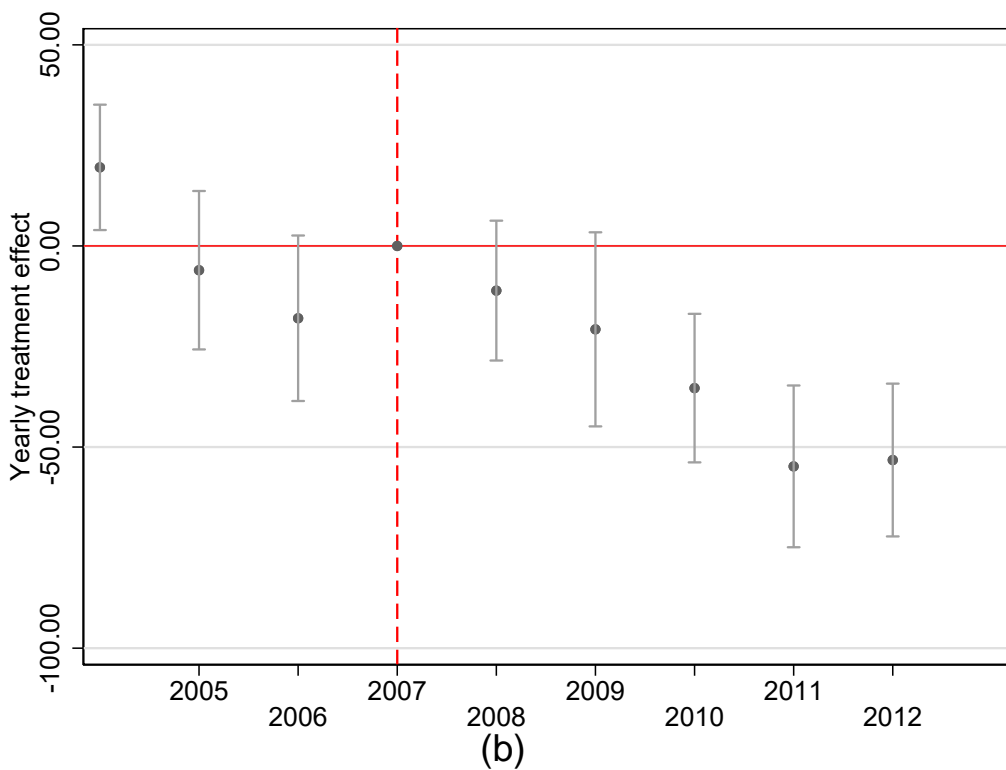

Supplement: S2 Fig — (PDF) [file pone.0334507.s003.pdf]

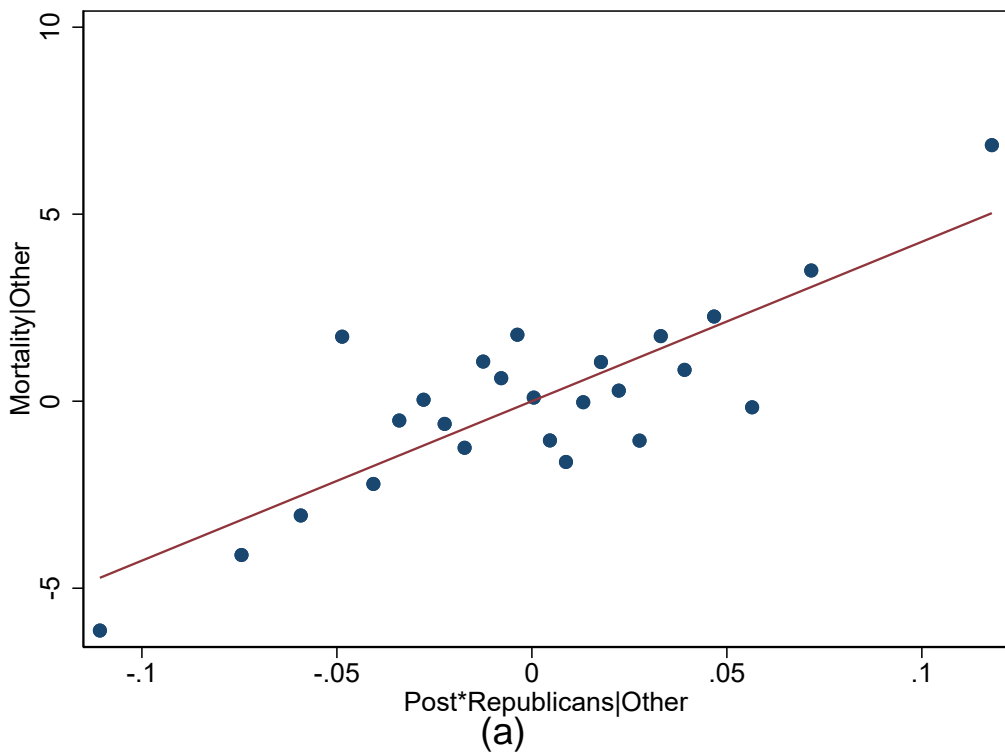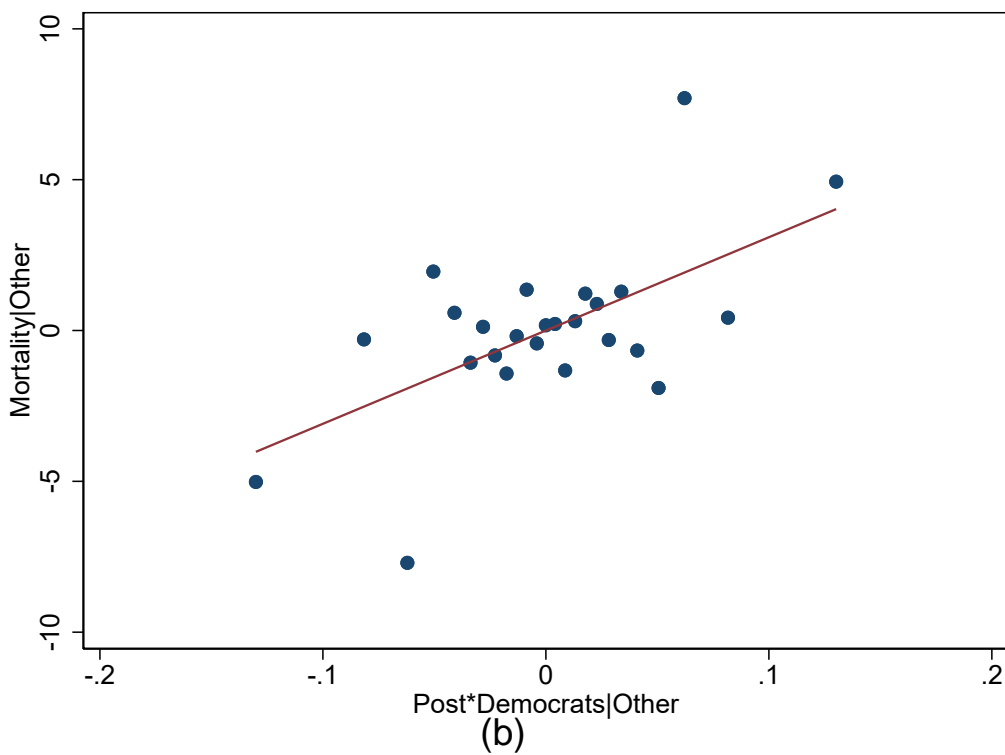

Supplement: S3 Fig — (PDF) [file pone.0334507.s004.pdf]

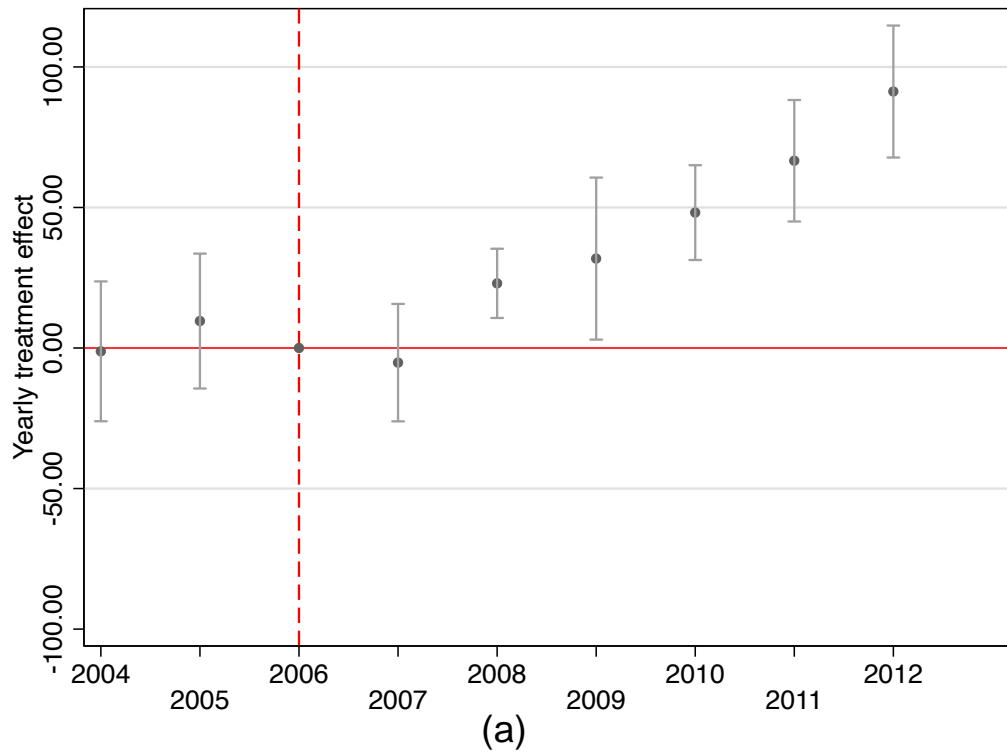

Supplement: S4 Fig — (PDF) [file pone.0334507.s005.pdf]

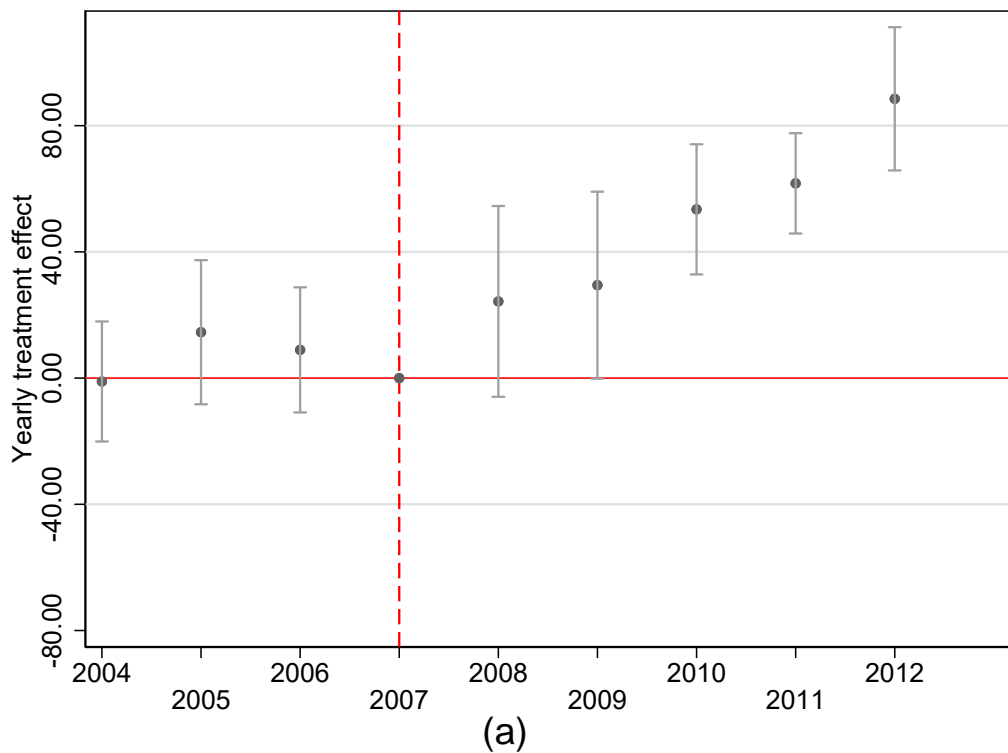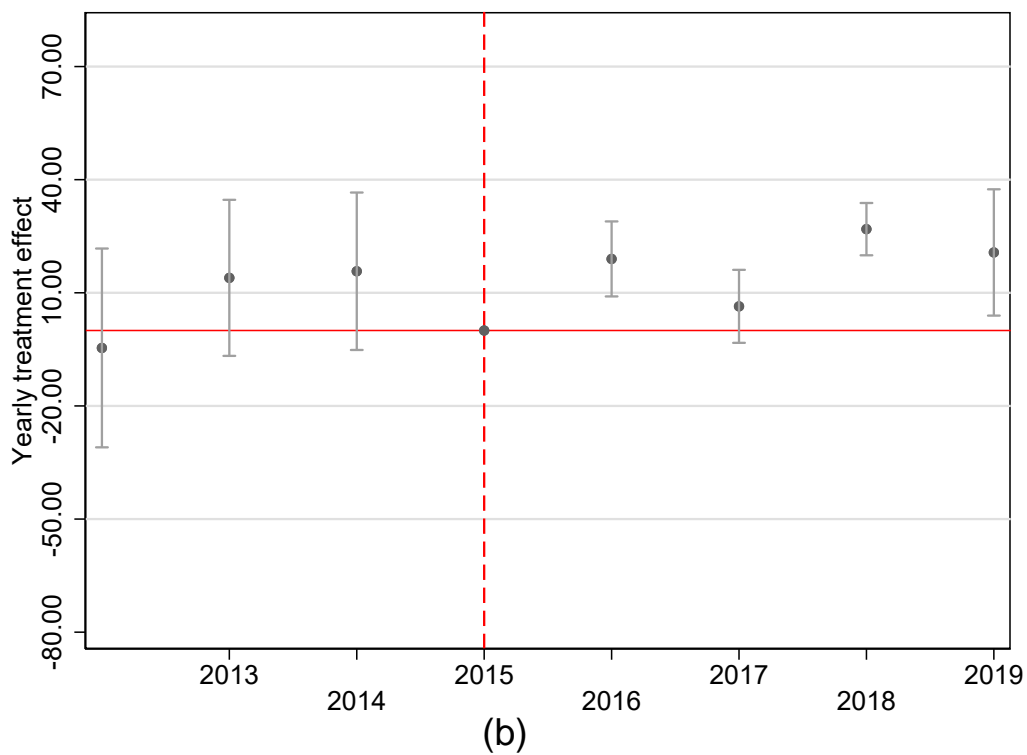

Supplement: S5 Fig — (PDF) [file pone.0334507.s006.pdf]

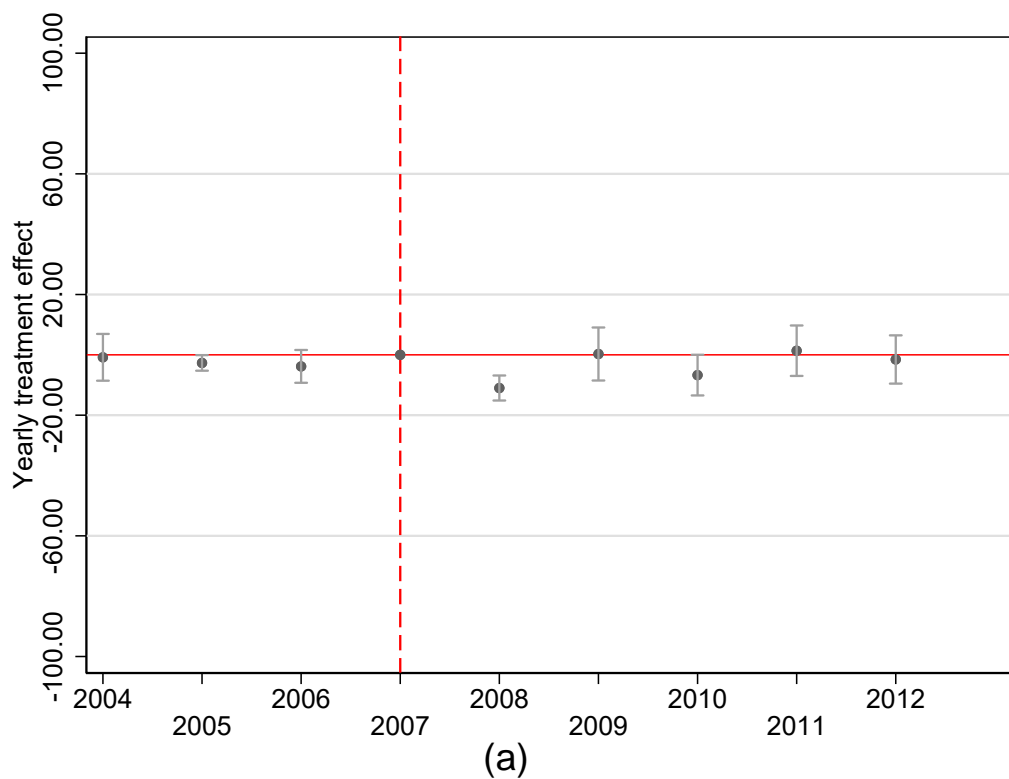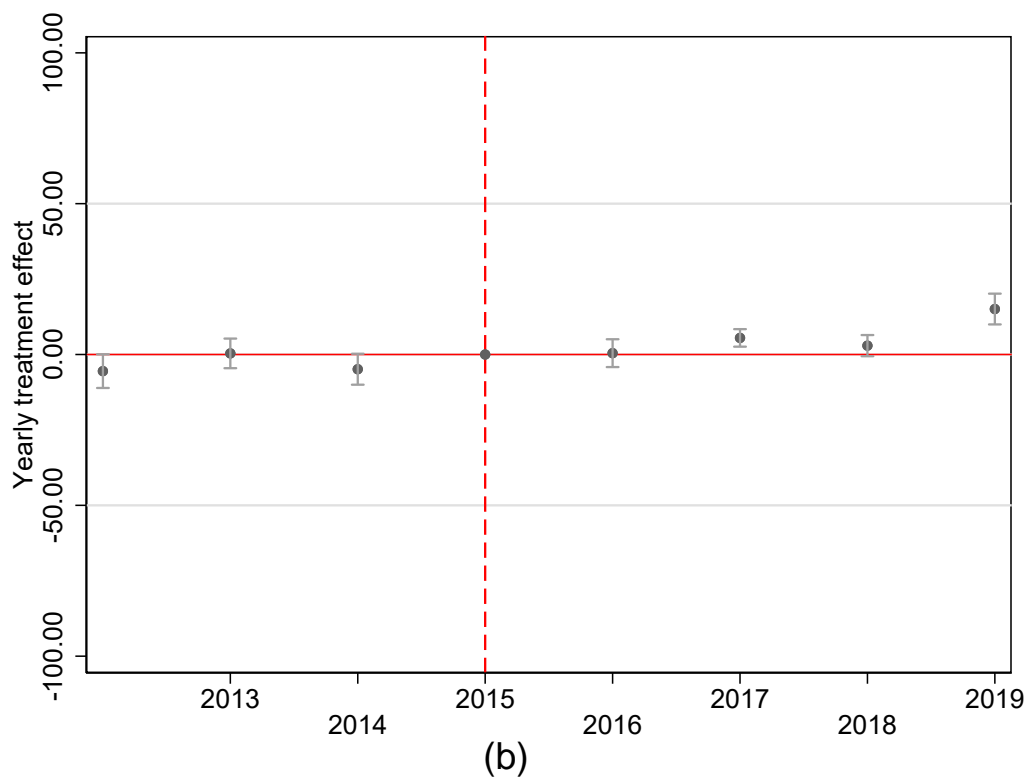

Supplement: S6 Fig — (PDF) [file pone.0334507.s007.pdf]

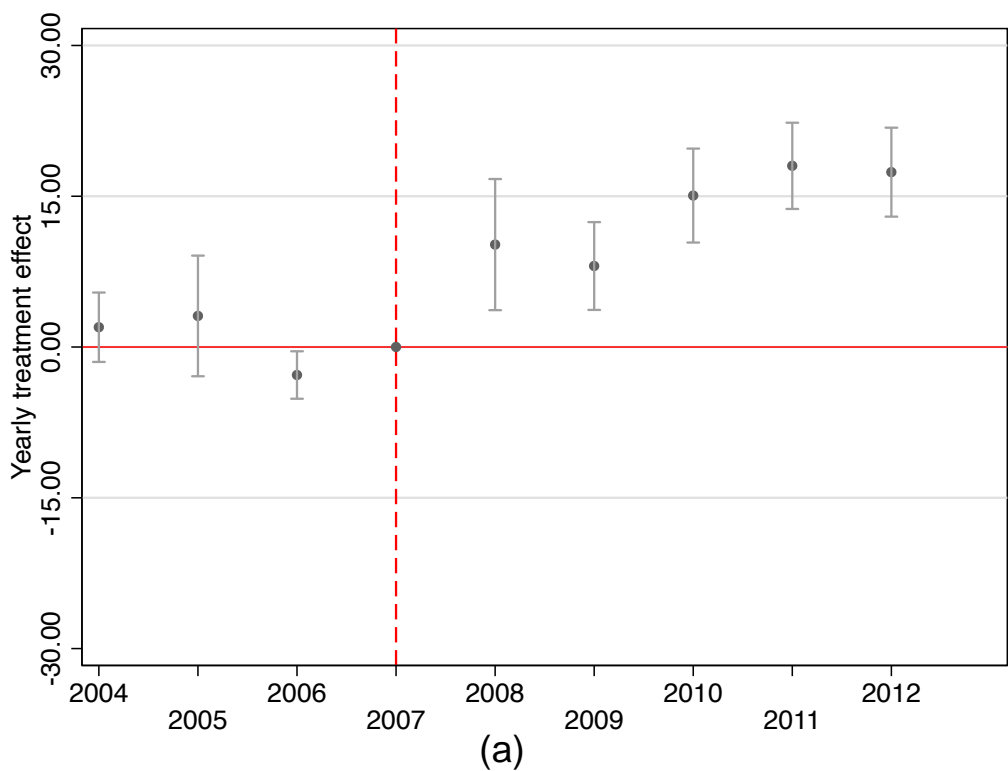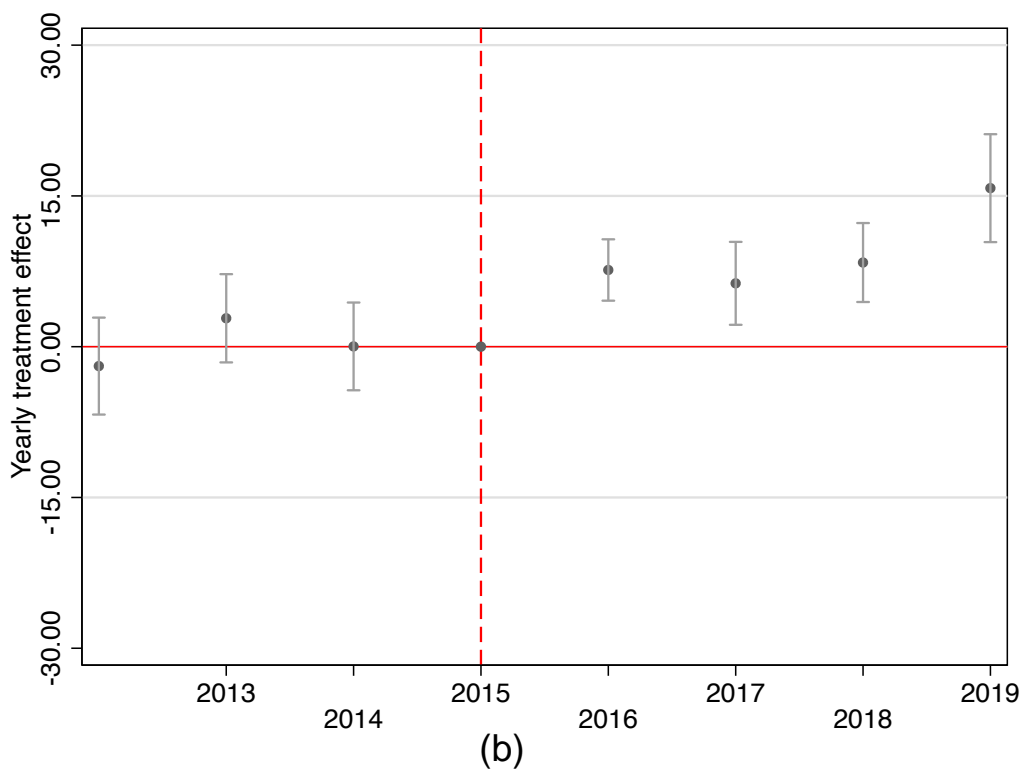

Supplement: S7 Fig — (PDF) [file pone.0334507.s008.pdf]
