# Supplementary material for: The health costs of losing political representation: Evidence from U.S. Presidential Elections
Source: PLoS One. 2025 Oct 31;20(10):e0334507. doi: 10.1371/journal.pone.0334507 (PMC12578145; doi:10.1371/journal.pone.0334507)
Supplement: S1 Table — (PDF) [file pone.0334507.s009.pdf]

Table S1: Population Weights

**Age Category**	Weights
0	0.0136657
1-4 years	0.0543033
5-14 years	0.1456663
15-24 years	0.1396771
25-34 years	0.1410695
35-44 years	0.160081
45-54 years	0.1346706
55-64 years	0.086578
65-74 years	0.0651537
75-84 years	0.0440284
85 years	0.0151064

Notes: The table shows population weights per age category that we use to build our age adjusted mortality rates.
