# Supplementary material for: The health costs of losing political representation: Evidence from U.S. Presidential Elections
Source: PLoS One. 2025 Oct 31;20(10):e0334507. doi: 10.1371/journal.pone.0334507 (PMC12578145; doi:10.1371/journal.pone.0334507)
Supplement: S2 Table — (PDF) [file pone.0334507.s010.pdf]

Table S2: Variable description

Variable name	Description	Source
**Panel A: County Variables**		
Mortality	Age-adjusted mortality rate per 100,000 county population. Rates are computed as a weighted average of the crude death rates across age categories within a county, where the shares of the overall U.S. population in each age category are used as weights	CDC
Democrats	The share of votes for the Democrat candidate during the 2016 Presidential election	MIT
Republicans	The share of votes for the Republican candidate during the 2008 Presidential election	MIT
Electoral Loss	Dummy variable equal to 1 if the county political preferences (using as threshold the median value of the Democrat variable distribution) is the same of the party of the President	MIT
Income	The natural logarithm of the county income per capita	BLS
Population	The natural logarithm of the county population	BLS
Unemployment	The county unemployment rate	BLS
Employment	The natural logarithm of the number of employees	CBP
Establishments	The natural logarithm of the number of establishments	CBP
Wages	The natural logarithm of the total annual payroll ($1,000)	CBP
Transfers	The natural logarithm of income payments to persons for which no current services are performed and net insurance settlements. It is the sum of government social benefits and net current transfer receipts from business	BLS
HPI	A weighted, repeat-sales index, that measures average price changes in repeat sales or refinancings on the same properties	FHFA
Polarization	The absolute difference between a county's political preferences for the Democratic party and the political preferences of the neighboring counties for the same party	MIT
Share Independent	The share of the votes for other parties in the Presidential elections	MIT
Share No Voters	The share of people in the county that did not vote in the Presidential election	MIT
Membership	The total number of membership associations divided by 10'000 population. We identify membership associations using the NAICS code (813410, 713950, 713910, 713940, 711211, 813110, 813940, 813930, 813910, and 813920)	NETS

Notes: This table shows a detailed description of each variable and its source.

Table S2: Variable description cont'd

**Panel B: BRFSS**		
General Health	A self-rated measure of general health measured considering the following question: *"Would you say that in general your health is excellent, very good, good, fair, poor?"*. We assigned a maximum value of 4 to "Excellent" and a minimum value of 0 to "Poor"	BRFSS
Mental Health	A self-rated measure of mental health measured considering the following question: *"Now thinking about your mental health, which includes stress, depression, and problems with emotions, for how many days during the past 30 days was your mental health not good?"*. The variable spans from a minimum of 0 to a maximum value of 30	BRFSS
Age	The age of the individuals replying to the survey	BRFSS
Income	Eight categorical variables for the category of the income of the individual	BRFSS
Female	Dummy variable equal to 1 if the respondent declares to be a female	BRFSS
Marital Status	Dummy variable equal to 1 if the respondent declares to be married	BRFSS
**Panel C: GSS**		
General Health	A self-rated measure of general health measured considering the following question: *"Would you say that in general your health is excellent, very good, good, fair, poor?"*. We assigned a maximum value of 4 to "Excellent" and a minimum value of 0 to "Poor"	GSS
Democrat	A self-rated measure of political identity measured considering the following question: *"Generally speaking, do you usually think of yourself as a Republican, Democrat, Independent, or what??"*. We assigned a value of 1 if the individual replied "Strong Democrat" and 0 otherwise	GSS
Republican President	A dummy variable equal to one if the president is a Republican	Wikipedia
Age	The age of the individuals replying to the survey	GSS
Income	Twelve categorical variables for the category of the income of the individual	GSS
Female	Dummy variable equal to 1 if the respondent declares to be a female	GSS
Marital Status	Dummy variable equal to 1 if the respondent declares to be married	GSS

Notes: This table shows a detailed description of each variable and its source.
