# Supplementary material for: The health costs of losing political representation: Evidence from U.S. Presidential Elections
Source: PLoS One. 2025 Oct 31;20(10):e0334507. doi: 10.1371/journal.pone.0334507 (PMC12578145; doi:10.1371/journal.pone.0334507)
Supplement: S3 Table — (PDF) [file pone.0334507.s011.pdf]

Table S3: Mental Health

Variables	(1) Mental Health > 0	(2) Mental Health > 0	(3) Log(Mental Health)
Post × Republicans	0.0377***	0.0413***	0.0709**
	(0.0113)	(0.0138)	(0.0274)
Age		0.0044***	0.0192***
		(0.0002)	(0.0005)
Age squared		-0.0001***	-0.0003***
		(0.0000)	(0.0000)
Married		-0.0397***	-0.0956***
		(0.0010)	(0.0025)
Female		0.0959***	0.1801***
		(0.0010)	(0.0022)
Income = 2		-0.0438***	-0.1725***
		(0.0020)	(0.0058)
Income = 3		-0.0831***	-0.3084***
		(0.0020)	(0.0067)
Income = 4		-0.1101***	-0.4081***
		(0.0023)	(0.0075)
Income = 5		-0.1419***	-0.5192***
		(0.0023)	(0.0072)
Income = 6		-0.1611***	-0.5939***
		(0.0025)	(0.0078)
Income = 7		-0.1806***	-0.6670***
		(0.0028)	(0.0085)
Income = 8		-0.2173***	-0.7681***
		(0.0030)	(0.0091)
County FE	Yes	Yes	Yes
Time FE	Yes	Yes	Yes
Observations	2,954,844	2,954,844	2,954,844
Adjusted R-squared	0.00462	0.0700	0.0785

Notes: This table shows regression results for Equation (**??**). *Mental Health* is the dependent variable and is a dummy variable equal to 1 if (or the natural logarithm of) the number of days in the last month the individual reported his/her mental health was not good is at least one. Mental health issues include stress, depression, and problems with emotions. Standard errors are double clustered at the county and time level. ***, **, and * denote significance at 1, 5, and 10 percent level respectively. See section **??** of the online appendix for a detailed description of every variable.
