# Supplementary material for: The health costs of losing political representation: Evidence from U.S. Presidential Elections
Source: PLoS One. 2025 Oct 31;20(10):e0334507. doi: 10.1371/journal.pone.0334507 (PMC12578145; doi:10.1371/journal.pone.0334507)
Supplement: S4 Table — (PDF) [file pone.0334507.s012.pdf]

Table S4: Normalized Differences

	Winning Counties		Losing Counties		
	Mean	SD	Mean	SD	ND
Income(log)	10.46	0.27	10.34	0.31	0.29
Population(log)	10.24	1.50	10.10	1.40	0.06
Unemployment(log)	6.94	2.89	5.12	2.21	0.50
Establishment(log)	6.41	1.52	6.30	1.41	0.05
Employment(log)	8.75	1.98	8.65	1.76	0.03
Wages(log)	12.22	2.13	12.01	1.97	0.07
HPI(log)	4.89	0.17	4.86	0.20	0.13
Trasnfers(log)	8.95	0.27	8.76	0.39	0.39

Notes: The table shows descriptive statistics for the county characteristics. We separate the sample into losing and winning counties. The last column provides normalized differences [?]. See section **??** of the online appendix for a detailed description of every variable.
