# Supplementary material for: The health costs of losing political representation: Evidence from U.S. Presidential Elections
Source: PLoS One. 2025 Oct 31;20(10):e0334507. doi: 10.1371/journal.pone.0334507 (PMC12578145; doi:10.1371/journal.pone.0334507)
Supplement: S5 Table — (PDF) [file pone.0334507.s013.pdf]

Table S5: Black Individuals

Variables	(1) Mortality	(2) Mortality	(3) Mortality
Post × Black	-35.1221**	-34.4535**	-35.9724**
	(10.6157)	(11.1740)	(11.5380)
County FE	Yes	Yes	Yes
Year FE	Yes	Yes	Yes
Race FE	Yes	Yes	Yes
Race-Year FE	No	Yes	Yes
State-Year FE	No	Yes	Yes
Post × County Controls	No	No	Yes
Observations	112,736	112,736	103,369
Adjusted R-squared	0.0978	0.151	0.150

Notes: This table shows regression results for equation (**??**). Mortality is the dependent variable and is the race-age-adjusted mortality rate. Standard errors are double clustered at the county and year level. ***, **, and * denote significance at 1, 5, and 10 percent level respectively. See section **??** of the online appendix for a detailed description of every variable.
