# Supplementary material for: The health costs of losing political representation: Evidence from U.S. Presidential Elections
Source: PLoS One. 2025 Oct 31;20(10):e0334507. doi: 10.1371/journal.pone.0334507 (PMC12578145; doi:10.1371/journal.pone.0334507)
Supplement: S6 Table — (PDF) [file pone.0334507.s014.pdf]

Table S6: Outliers

Variables	(1) Mortality	(2) Mortality	(3) Mortality
Panel A: Obama and Mortality			
Post × Republicans	41.8002***	45.9572***	41.2698**
	(9.0094)	(12.0122)	(13.6534)
County FE	Yes	Yes	Yes
Year FE	Yes	Yes	Yes
State-Year FE	No	Yes	Yes
Post × Controls	No	No	Yes
Observations	28,017	28,017	27,540
Adjusted R-squared	0.730	0.735	0.733
Panel B: Trump and Mortality			
Post × Democrats	17.9666*	21.4956*	23.9830**
	(9.1956)	(9.8313)	(10.1013)
County FE	Yes	Yes	Yes
Year FE	Yes	Yes	Yes
State-Year FE	No	Yes	Yes
Fully interacted controls	No	No	Yes
Observations	24,912	24,904	24,480
Adjusted R-squared	0.765	0.766	0.765

Notes: This table shows regression results for equation (**??**). *Mortality* is the dependent variable and is the age-adjusted mortality rate of the county. We winsorize all the variables at the first and last percentiles. Standard errors are double-clustered at the county level. ***, **, and * denote significance at 1, 5, and 10 percent level respectively. See section **??** of the online appendix for a detailed description of every variable.
