# Supplementary material for: The health costs of losing political representation: Evidence from U.S. Presidential Elections
Source: PLoS One. 2025 Oct 31;20(10):e0334507. doi: 10.1371/journal.pone.0334507 (PMC12578145; doi:10.1371/journal.pone.0334507)
Supplement: S7 Table — (PDF) [file pone.0334507.s015.pdf]

Table S7: Clustering

Variables	(1) Mortality	(2) Mortality	(3) Mortality	(4) Mortality
Panel A: Obama and Mortality				
Post × Republicans	44.6786***	44.6786**	44.6786**	44.6786**
	(14.8596)	(15.2342)	(19.7121)	(18.4464)
County FE	Yes	Yes	Yes	Yes
Year FE	Yes	Yes	Yes	Yes
State-Year FE	Yes	Yes	Yes	Yes
Post × Controls	Yes	No	Yes	Yes
Cluster	County	County and Year	State	State and Year
Observations	27,540	27,540	27,540	27,540
Adjusted R-squared	0.703	0.703	0.703	0.703
Panel B: Trump and Mortality				
Post × Democrats	30.9178**	30.9178**	30.9178**	30.9178*
	(14.2460)	(12.3927)	(15.3085)	(13.1430)
County FE	Yes	Yes	Yes	Yes
Year FE	Yes	Yes	Yes	Yes
State-Year FE	Yes	Yes	Yes	Yes
Post × Controls	Yes	No	Yes	Yes
Cluster	County	County and Year	State	State and Year
Observations	24,480	24,480	24,480	24,480
Adjusted R-squared	0.747	0.747	0.747	0.747

Notes: This table shows regression results for equation (**??**) when we cluster the standard errors at different level. *Mortality* is the dependent variable and is the age-adjusted mortality rate of the county. ***, **, and * denote significance at 1, 5, and 10 percent level respectively. See section **??** of the online appendix for a detailed description of every variable.
