# Supplementary material for: The health costs of losing political representation: Evidence from U.S. Presidential Elections
Source: PLoS One. 2025 Oct 31;20(10):e0334507. doi: 10.1371/journal.pone.0334507 (PMC12578145; doi:10.1371/journal.pone.0334507)
Supplement: S8 Table — (PDF) [file pone.0334507.s016.pdf]

Table S8: The Great Recession

Variables	(1) Mortality	(2) Mortality	(3) Mortality
Post × Republicans	43.0299***	49.3701***	44.9463**
	(11.5373)	(13.8752)	(15.5446)
Post × Severity	-17.9114**	-8.9031	-2.5961
	(5.5928)	(6.4375)	(7.0448)
County FE	Yes	Yes	Yes
Year FE	Yes	Yes	Yes
State-Year FE	No	Yes	Yes
County controls	No	No	Yes
Observations	28,008	28,008	27,540
Adjusted R-squared	0.700	0.705	0.703

Notes: This table shows regression results for equation (**??**) and we control for the severity of the Great Recession. *Mortality* is the dependent variable and is the age-adjusted mortality rate of the county. Standard errors are double clustered at the county and year level. ***, **, and * denote significance at 1, 5, and 10 percent level respectively. See section **??** of the online appendix for a detailed description of every variable.
