# Supplementary material for: The health costs of losing political representation: Evidence from U.S. Presidential Elections
Source: PLoS One. 2025 Oct 31;20(10):e0334507. doi: 10.1371/journal.pone.0334507 (PMC12578145; doi:10.1371/journal.pone.0334507)
Supplement: S9 Table — (PDF) [file pone.0334507.s017.pdf]

Table S9: Placebo tests

Variables	(1)<br>Mortality	(2)<br>Mortality	(3)<br>Mortality	(4)<br>Mortality
Panel A: Obama				
Post × Share Republicans (Baseline)	6.169***			
	(2.052)			
Post × Share No Voters		5.222		
		(3.248)		
Post × Share Independent			1.358	
			(3.115)	
Post × Share Republicans (Placebo)				3.013
				(1.969)
County FE	Yes	Yes	Yes	Yes
Year FE	Yes	Yes	Yes	Yes
State-Year FE	Yes	Yes	Yes	Yes
County controls	Yes	Yes	Yes	Yes
Observations	27,540	27,540	27,540	22,856
Adjusted R-squared	0.703	0.703	0.703	0.768
Panel B: Trump				
Post × Share Democrats (Baseline)	4.706**			
	(1.886)			
Post × Share Did not Vote		1.401		
		(1.872)		
Post × Share Independent			0.631	
			(3.821)	
Post × Share Democrats (Placebo)				-2.954
				(1.983)
County FE	Yes	Yes	Yes	Yes
Year FE	Yes	Yes	Yes	Yes
State-Year FE	Yes	Yes	Yes	Yes
County controls	Yes	Yes	Yes	Yes
Observations	24,480	24,480	24,480	22,856
Adjusted R-squared	0.747	0.747	0.747	0.768

Notes: This table shows regression results for Equation (**??**) when we consider alternative measures of county political preferences and a placebo test base on the Presidential election of 2012. *Mortality* is the dependent variable and is the age-adjusted mortality rate of the county. ***, **, and * denote significance at 1, 5, and 10 percent level respectively. See section **??** of the online appendix for a detailed description of every variable.
