# Supplementary material for: The health costs of losing political representation: Evidence from U.S. Presidential Elections
Source: PLoS One. 2025 Oct 31;20(10):e0334507. doi: 10.1371/journal.pone.0334507 (PMC12578145; doi:10.1371/journal.pone.0334507)
Supplement: S10 Table — (PDF) [file pone.0334507.s018.pdf]

Table S10: General Health

Variables	(1)<br>General Health	(2)<br>General Health
Post Republicans	-0.0328*	-0.0432***
	(0.0167)	(0.0129)
Age		-0.0287***
		(0.0006)
Age squared		0.0002***
		(0.0000)
Married		-0.0393***
		(0.0024)
Female		0.0865***
		(0.0023)
Income = 2		0.1471***
		(0.0059)
Income = 3		0.3496***
		(0.0077)
Income = 4		0.5328***
		(0.0091)
Income = 5		0.7268***
		(0.0084)
Income = 6		0.9118***
		(0.0092)
Income = 7		1.0800***
		(0.0097)
Income = 8		1.3117***
		(0.0097)
County FE	Yes	Yes
Time FE	Yes	Yes
Observations	2,929,186	2,929,186
Adjusted R-squared	0.0294	0.184

Notes: This table shows regression results for Equation (**??**). *General Health* is the dependent variable. It is a measure of general health status that we obtain considering the following question: *"Would you say that in general your health is excellent, very good, good, fair, poor?"*. We assigned a maximum value of 4 to "Excellent" and a minimum value of 0 to "Poor" . Standard errors are double clustered at the county and time level. ***, **, and * denote significance at 1, 5, and 10 percent level respectively. See section **??** of the online appendix for a detailed description of every variable.
