# Supplementary material for: The health costs of losing political representation: Evidence from U.S. Presidential Elections
Source: PLoS One. 2025 Oct 31;20(10):e0334507. doi: 10.1371/journal.pone.0334507 (PMC12578145; doi:10.1371/journal.pone.0334507)
Supplement: S11 Table — (PDF) [file pone.0334507.s019.pdf]

Table S11: The GSS

Variables	(1)<br>Health	(2)<br>Health	(3)<br>Health
Democrat	-0.0964***	-0.0832***	0.0015
	(0.0162)	(0.0162)	(0.0154)
Republican President × Democrat	-0.0650***	-0.0705***	-0.0353*
	(0.0225)	(0.0225)	(0.0211)
Republican President	0.0337***		
	(0.0086)		
Female			0.0047
			(0.0076)
Age			-0.0232***
			(0.0013)
Age Squared			0.0001***
			(0.0000)
Married			0.0364***
			(0.0083)
Income = 2			-0.0897**
			(0.0453)
Income = 3			-0.1412***
			(0.0461)
Income = 4			-0.0301
			(0.0472)
Income = 5			0.0141
			(0.0460)
Income = 6			0.0221
			(0.0456)
Income = 7			0.1047**
			(0.0448)
Income = 8			0.1137***
			(0.0411)
Income = 9			0.2158***
			(0.0375)
Income = 10			0.2938***
			(0.0379)
Income = 11			0.3665***
			(0.0379)
Income = 12			0.5874***
			(0.0363)
Constant	2.0357***		
	(0.0060)		
Region FE	No	Yes	Yes
Year FE	No	Yes	Yes
Other controls	No	No	Yes
Observations	43,943	43,943	43,943
R-squared	0.0038	0.0133	0.1198

Notes: This table shows regression results for Equation (**??**). *General Health* is the dependent variable. It is a measure of general health status that we obtain considering the following question: *“Would you say that in general your health is excellent, very good, good, fair, poor?”*. We assigned a maximum value of 4 to “Excellent” and a minimum value of 0 to “Poor”. Standard errors are adjusted for heteroskedasticity. ***, **, and * denote significance at 1, 5, and 10 percent level respectively. See section **??** of the online appendix for a detailed description of every variable.
