# Supplementary material for: The health costs of losing political representation: Evidence from U.S. Presidential Elections
Source: PLoS One. 2025 Oct 31;20(10):e0334507. doi: 10.1371/journal.pone.0334507 (PMC12578145; doi:10.1371/journal.pone.0334507)
Supplement: S12 Table — (PDF) [file pone.0334507.s020.pdf]

Table S12: Additional control variables

Variables	(1) Mortality	(2) Mortality
Post × Republicans	39.184**	
	(14.901)	
Post × Democrats		32.880**
		(12.199)
County FE	Yes	Yes
Year FE	Yes	Yes
State-Year FE	Yes	Yes
Interacted controls + Addtional controls	Yes	Yes
Observations	27,531	24,472
Adjusted R-squared	0.703	0.747

Notes: This table shows regression results for Equation (**??**). *Mortality* is the dependent variable and is the age-adjusted mortality rate in the county. We additional control for: a) the median age in the county, b) the percentage with a BA or higher, c) the percentage with health insurance in each county, and d) a dummy variable equal to one if the county is a minority county. Standard errors are double clustered at the county and year level. ***, **, and * denote significance at 1, 5, and 10 percent level respectively. See section **??** of the online appendix for a detailed description of every variable.
