# Supplementary material for: The health costs of losing political representation: Evidence from U.S. Presidential Elections
Source: PLoS One. 2025 Oct 31;20(10):e0334507. doi: 10.1371/journal.pone.0334507 (PMC12578145; doi:10.1371/journal.pone.0334507)
Supplement: Attachment — Submitted filename: Reply to the referees .pdf [file pone.0334507.s021.pdf]

Revision of the manuscript "Partisan Health"

Dear Referee,

We would like to thank you very much for your thoughtful and constructive feedback, which helped us to improve the paper substantially.

Before addressing the specific points you raised, we would like to present an overview of the significant modifications made in the revised version. More specifically, the key changes can be summarized as follows:

- We have explained more clearly how our paper contributes to the existing literature, and we have provided a more detailed comparison between our paper and related existing studies.

- We discuss in more detail and test the parallel trend assumption and its validity in the context of our paper.

- We explain more clearly the theoretical background behind our findings.

In the remainder, we put your comments and suggestions in *italics*, followed by our detailed response.

With best regards,

The authors

Comments to the author

The paper examines whether a change in political leadership impacts health outcomes. Specifically, the researchers analyze the impact of elections where power shifts, causing individuals to either align with or lose alignment with the party of the President. The findings indicate that a lack of political alignment has an immediate and long-lasting negative effect on health. The study finds no evidence that these effects can be explained by other confounding trends, economic changes, or policy shifts. Furthermore, the results suggest that political sentiments and social isolation play a role, and that the lack of political representation affects individuals' mental health.

The main question is relevant, and I find the manuscript very well written, but the paper should better explain how the current research contributes to the literature that is closest to the analysis and the research question. Also, I believe the authors can do more and discuss more in relation to key assumptions and recent developments of the DiD literature. Taken together my recommendation is to allow the authors to revise the paper for possible publication in PLOS One.

Reply: Thank you very much for your assessment of our paper. We hope you find the revised version clearer regarding its contribution to the literature and how it differs from previous studies. Additionally, we hope you find the expanded discussions on the key assumptions behind the validity of our empirical approach, as well as the additional results supporting its validity, to be adequate.

COMMENTS

CONTRIBUTION

I think the overview of the literature is ok but would prefer that the authors are more to the point. It seems to me there are some existing works that are mentioned in the manuscript that relate very closely to the work in this study, and they should be elaborated more upon and how this piece contributes more in detail in relation to these papers.

Reply: Thank you for your comment. In the revised submission, we have provided a clearer explanation of how our paper differs from the cited works in the final paragraph

of the introduction. Specifically, we have added the following comments.

Maas and Lu (2021) identified a positive correlation between mortality rates and partisan losses. In a similar vein, Wasfy et al. (2017) and Goldman et al. (2019) use regression analysis to demonstrate a correlation between health metrics and voting patterns, with voting outcomes serving as the dependent variable. Our study differs from these works by providing causal, unidirectional estimates of the impact of a shift in political power on mortality, leveraging turnover elections as natural experiments. In addition to this, our unique county-race-year level analysis of black mortality following Obama's election provides a within-county comparison that controls for unobserved county characteristics, which is a methodological improvement over studies that rely solely on cross-county comparisons. More importantly, we investigate the specific mechanisms driving these effects, emphasizing the role of political sentiments and social isolation while ruling out changes in economic characteristics as a potential channel. Overall, our analysis offers a more comprehensive and precise understanding of the relationship between health outcomes and political leadership.

Pierce et al. (2016) show that elections affected the happiness of partisan losers, but this effect dissipated within a week. Our study demonstrates that health effects are not transient but persist over years, suggesting a different and more concerning mechanism than temporary emotional responses. Furthermore, our long-term GSS analysis from 1973 provides historical context missing from most contemporary studies, offering evidence that the health-politics relationship has been consistent across multiple administrations.

While Mian et al. (2021) and Barsky and Sims (2012) focused on economic sentiments and consumption following political leadership changes, our paper examines both economic and non-economic pathways, showing that social isolation and mental health deterioration drive effects even in the absence of economic changes. We test for both sudden causes of death and chronic conditions, providing evidence for both immediate stress responses and cumulative effects, which is a more comprehensive approach than studies focusing on single pathways.

Our paper directly addresses the contradictory findings of Classen and Dunn (2010)

by suggesting that the benefits of social integration they observed in 1981-2005 may have been outweighed by increasing polarization in your more recent study period. We connect seemingly disparate literature streams (political polarization, social capital, and public health) in a coherent framework that helps explain why some previous studies found effects while others did not.

Unlike studies focusing on specific health behaviors (like Milosh et al. (2021) on mask-wearing), our paper quantifies the overall mortality burden of political misalignment, making it more relevant for broad public health policy. Our calculation of economic costs using FEMA's value of statistical life provides a concrete policy-relevant metric absent from most previous research on partisan health differences.

IDENTIFICTAION AND POTENTIAL THREATS

(a) PARALELL TREND ASSUMPTION

The authors show graphically that there are reasons to believe that the parallel trend assumption holds for overall mortality. I think it is important to also who this for specific death causes as this is also a focus in the analysis. Similarly, I am not sure the authors present graphs for specific groups examined.

Reply: We thank you for this input. In the revised version of the paper, we present graphs supporting the parallel trend assumption for the following outcome variables and events:

- Overall mortality rates in Democratic and Republican counties after the turnover presidential elections of Presidents Trump and Obama, respectively.
- Black morality rates after the turnover election of President Obama.
- Quarterly mortality rates for sudden causes of death for the turnover presidential elections of Presidents Trump and Obama, respectively.

We report all the graphs in Figures (Referee 1-1)-(Referee 1-4), as well as in the re-

vised version of the paper. More specifically, these results show that before a turnover in the Presidential elections, mortality rates are similar between Democratic and Republican counties, but they diverge afterward depending on the outcome of the election.

Figure Referee 1-2: Yearly treatment coefficients - Baseline specification

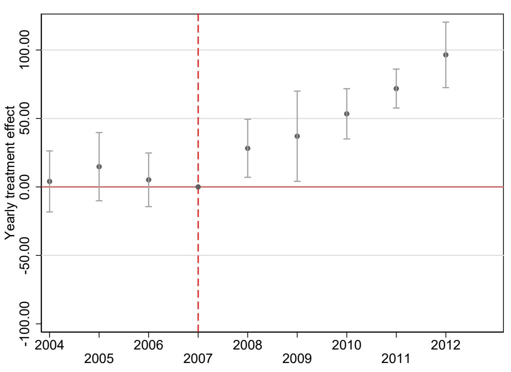

Referee 1-1 Obama and the Republicans

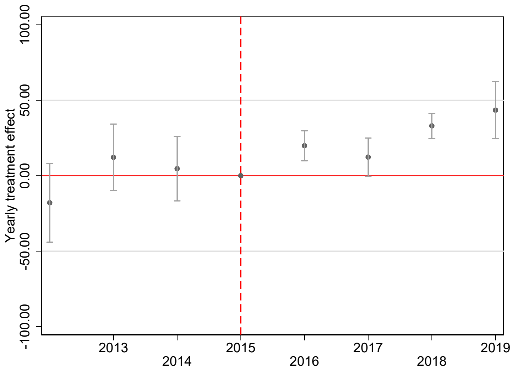

Referee 1-2 Trump and the Democrats

Figure Referee 1-3: Obama and black mortality rates

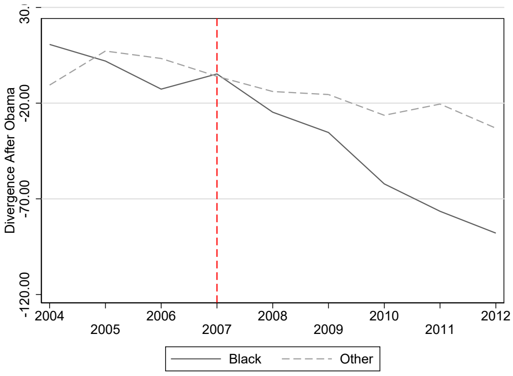

Referee 1-1 Parallel trends

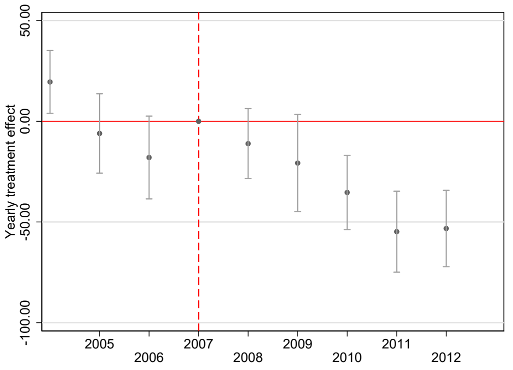

Referee 1-2 Event study analysis

Figure Referee 1-4: Political sentiments

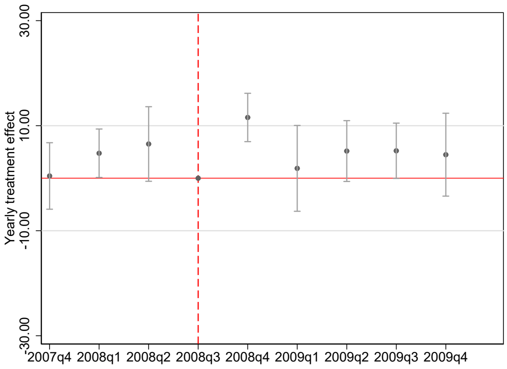

Referee 1-1 Obama and the Democrats

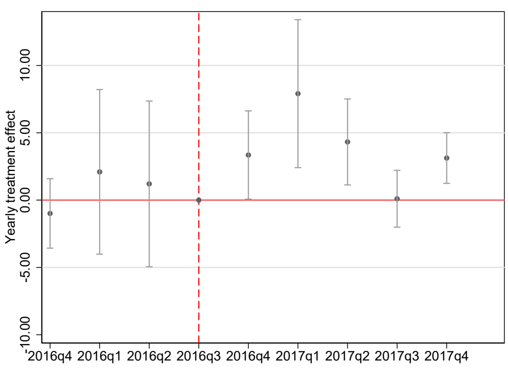

Referee 1-2 Trump and the Republicans

In an additional exercise reported in a paragraph in sub-section 2.4 in the revised version of the paper, we analyze the long-term dynamic effects of internal and external causes of death—the two primary broad categories defining mortality. The results are presented in Figures (Referee 1-5) and (Referee 1-6). We still find that the assumption of parallel trends is likely to hold since none of the coefficients is significant before the treatment. Additionally, our analysis shows that internal causes of death are the primary determinants of our results. On the other hand, the coefficients related to external causes of death are all close to zero even after turnover elections.

Figure Referee 1-5: Internals

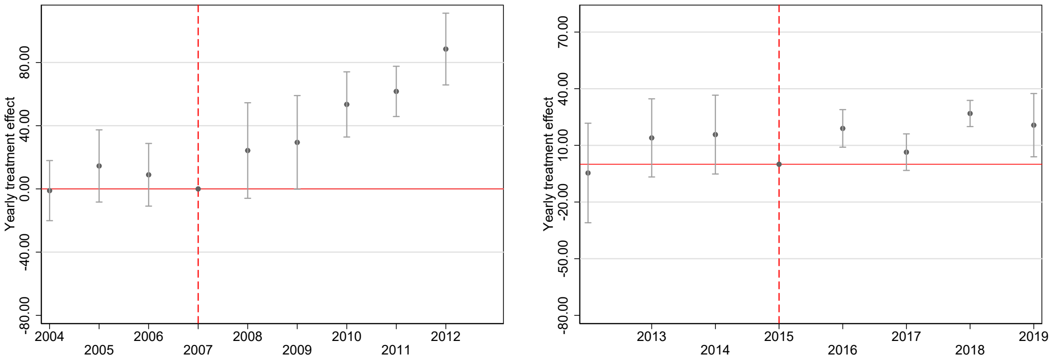

Referee 1-1 Obama and the Republicans

Referee 1-2 Trump and the Democrats

Figure Referee 1-6: External

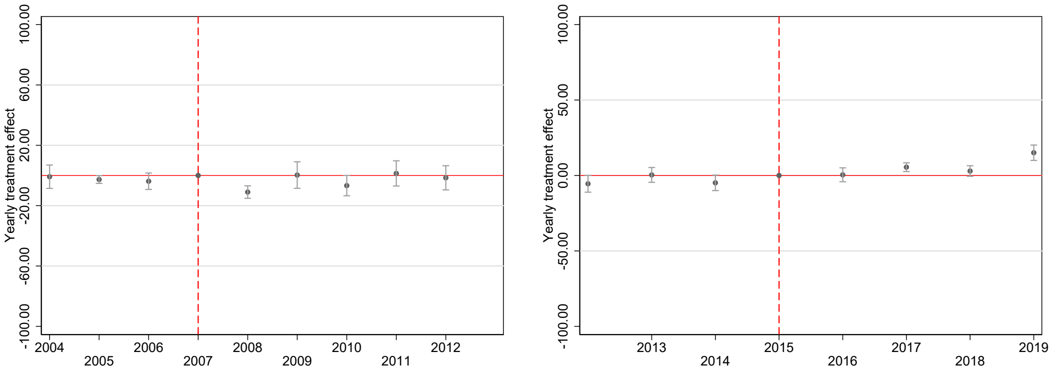

Referee 1-1 Obama and the Republicans

Referee 1-2 Trump and the Democrats

Also, I think it is relevant to discuss the likeliness of the parallel trend assumption to hold in relation to previous academic work showing that there are health effects before elections/shifts in power.

Reply: Thank you for this point. You raise an important point about potential pre-election health effects. Indeed, our analysis shows increased mortality in politically polarized counties before Presidential elections, consistent with Panagopoulos and Weinschenk (2022) who document deteriorating mental health during campaign periods. However, we discuss that these pre-election effects do not invalidate our difference-in-differences approach for two reasons:

- First, our time series graphs show that the mortality trends in Democratic and Republican counties remain parallel prior to the election. We provide a more detailed discussion of Figure (1) in our paper in response to your next question.
- Second, while campaign stress affects all counties, its elevation is likely to be similar across partisan counties before the election, with divergence only occurring after the election outcome is known.

We have enhanced our discussion in sub-section 2.2 in the revised version of the paper to address these considerations explicitly.

And finally, in Figure 1, what is it that we see in figure (a) between 2006 and 2007?

Reply: Thank you for this remark. In Figure 1(a) in the paper, there is a noticeable dip in mortality trends for both Democratic and Republican counties between 2006 and 2007, before the election of Obama. This could reflect a broader national improvement in health outcomes affecting all counties (for example, due to aggregate policy changes or demographic shifts). However, it is also important to note that a more substantial decrease in the mortality rate occurs in Democratic counties.

To test whether this difference is statistically significant and whether it leads to a violation of the parallel trends assumption, we re-estimated Equation (3) in the paper using

2006 as the reference year. The results are reported in Figure (Referee 1-7). We find that the interaction coefficient between the indicator variable for the year 2007 and the share of Democrats is not statistically significant and close to zero. Therefore, the greater decrease in mortality in Democratic counties before the election is unlikely to affect our findings or violate the assumption of parallel trends. We have commented on this result in a paragraph in sub-section 2.4 of the revised paper.

Figure Referee 1-7: Parallel trend assumption

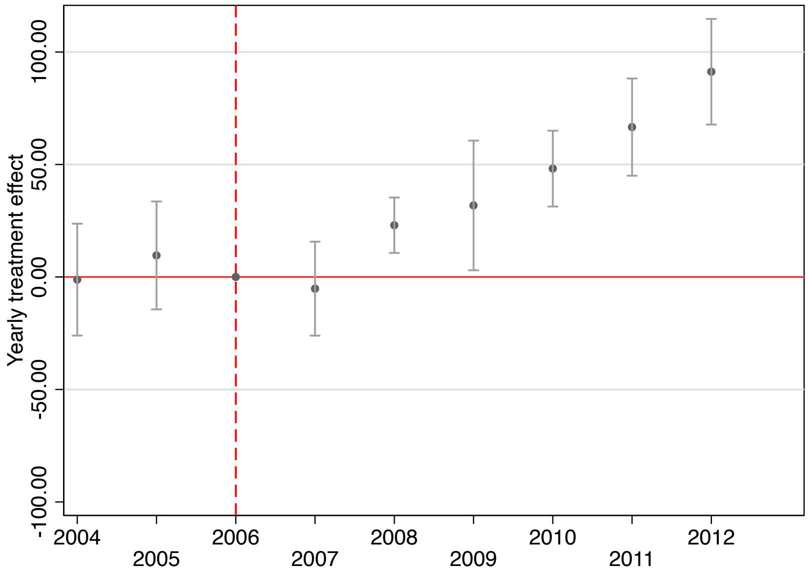

(b) IDENTIFICATION

In my view the causal claims should be restricted to the analysis regarding mortality. It is good that the authors try to take recent developments of the DiD method into account, but I would prefer more of elaboration regarding the potential identification problem that they have in the main specification/results and what is not fully solved by the suggested solution by Dahl et al (2022).

Reply: We agree that our causal claims should be focused on the mortality analysis, where our identification strategy is most robust. In the revised manuscript, we have clarified the distinction between our causal findings on mortality and the more suggestive

evidence regarding mechanisms. We have adjusted our language accordingly throughout to ensure we do not overstate causal interpretations beyond what our identification strategy supports.

Regarding your concern about the difference-in-differences implementation, we have expanded our discussion of potential identification challenges in Section 5. In addition, while we follow Dahl et al. (2022) in using threshold-based binary treatment assignment as a robustness check, we acknowledge this does not fully address all identification concerns with continuous treatment variables highlighted in recent econometric literature (Callaway et al., 2024). More specifically, we explicitly discussed the limitations of our continuous treatment approach and clarified that, although our new exercise is suggestive and supports our causality claims of an effect of turnover elections on the health of the individuals, it does not guarantee that our estimators are unbiased. In fact, our new exercise does not ensure that the strong parallel trends assumption holds for every treatment level.

We believe these additions in a paragraph in sub-section 2.4 in the revised version of the paper substantially strengthen our paper by being transparent about identification challenges while demonstrating that our core findings on mortality effects remain robust across alternative specifications and sensitivity analyses. Thank you again for pushing us to improve the rigor of our analysis.

ARGUMENTATION

The hypothesis to why political sentiments as a mechanism would correlate with sudden deaths is not well motivated. I think the argumentation must be strengthened and related to previous literature on this point.

Reply: Thank you for this important observation. We acknowledge that our explanation of why political sentiments would correlate with sudden deaths requires stronger motivation and connection to established literature. In the revised manuscript, we have substantially expanded subsection 2.3.1 with the following comments.

First, we have strengthened the theoretical foundation by drawing on the extensive stress-health literature. Acute psychological stressors trigger well-documented physiological responses, including increased cardiovascular reactivity, inflammatory responses, and dysregulation of the hypothalamic-pituitary-adrenal axis (McEwen, 1998; Kivimäki and Steptoe, 2018). These mechanisms provide biological pathways connecting sudden political disappointment to acute health events.

Second, we have incorporated research specifically on political events and acute health outcomes. We cite Rosman et al. (2021), who documented increased arrhythmia risk during the 2016 presidential election, and Mefford et al. (2020), who found elevated cardiovascular hospitalizations around the same period.

Third, we connect our findings to the concept of ”broken heart syndrome” (takotsubo cardiomyopathy), where sudden emotional stress can trigger cardiovascular events even in otherwise healthy individuals (Templin et al., 2015). This literature provides a medical mechanism for how election-related distress could manifest as sudden mortality.

With these additions, we believe our argument for political sentiments as a mechanism for sudden deaths is now much better grounded in both theoretical frameworks and empirical evidence from multiple disciplines.

DATA

Is it noted in the death certificate data if a person has a different nationality or not recorded in a census? I think there are reasons to believe that e.g. that people in the US illegally would be more stressed when there are political shifts of different kinds as the ones examined here. Is there a possibility to sort out the death rates among non-US citizens dying in the counties?

Reply: Thank you for raising your question regarding nationality in our mortality data. In the US, death certificates record an individual's residence status (i.e., whether the person resides outside the U.S.) and the decedent's place of birth, which can indicate a foreign birth.

While your point about differential stress among non-citizens during political tran-

sitions is well-founded and relevant, there are significant limitations to addressing this directly with our data. For what concerns the residence information, only 0.1% of the deceased individuals have a residence in a foreign country. On the other hand, regarding the birthplace information, a potential problem with this variable is that it does not distinguish between legal permanent residents, naturalized citizens, visa holders, or undocumented immigrants for people that have born outside the United States.

In response to your suggestion, we conducted a robustness check by performing an additional analysis using variable mortality rates for foreign-born individuals as the outcome variable and re-estimating our main models. However, as reported in Table Referee 1-1, we found no significant effects of turnover elections on the health of these individuals. This is likely due to measurement errors that bias our coefficients of interest toward zero.

Table Referee 1-1: Difference-in-differences

**Variables**	(1) Mortality	(2) Mortality	(3) Mortality
Panel A: Obama and Mortality			
Post × Republicans	-31.0742*	0.4559	0.7949
	(14.4510)	(4.1565)	(5.1819)
County FE	Yes	Yes	Yes
Year FE	Yes	Yes	Yes
State-Year FE	No	Yes	Yes
Interacted controls	No	No	Yes
Observations	27,972	27,963	27,486
Adjusted R-squared	0.415	0.950	0.950
Panel B: Trump and Mortality			
Post × Democrats	-4.7436	-1.4860	3.0840
	(2.7512)	(2.4743)	(4.2943)
County FE	Yes	Yes	Yes
Year FE	Yes	Yes	Yes
State-Year FE	No	Yes	Yes
Interacted controls	No	No	Yes
Observations	24,856	24,856	24,432
Adjusted R-squared	0.909	0.911	0.912

I am not very fond of the use of the membership association data. Rather than the number of organizations it should focus on the number of people being members in associations. I am sure this is reported in the NETS database.

Reply: Thank you for your input. Unfortunately, this information is not available in the NETS database. The database is designed to track the dynamics of the US economy. However, it remains useful for our purposes, as it provides data on the dynamics of the universe of establishments in the United States, including nonprofit organizations.

In addition to the characteristics of the establishments, the database also reports an estimate of the number of employees. We sum this value over all the establishments in a county and year and divide it by the county population. Next, we use this variable as an outcome variable in Equation (3) in the paper.

The coefficients of interest — the interaction between the post period and the share of Republicans in the Obama setting and the share of Democrats in the Trump setting — are consistently negative but not statistically significant at the conventional 10% level, with reported p-values of 0.102 and 0.190, respectively. A likely explanation for the lack of statistically significant results is that, while the NETS database is useful for tracking the presence of establishments over time, its employee counts are often estimated (particularly for small businesses) and static, and are therefore not recommended for use in a dynamic analysis (Barnatchez et al., 2017).

Is the BRFSS data representative at the county level?

Reply: The Behavioral Risk Factor Surveillance System (BRFSS) is not designed to be representative at the county level. It uses state-based sampling designed to produce estimates that are representative at the state level, with some metropolitan/micropolitan statistical areas also having sufficient sample sizes for reliable estimates.

In our paper, we acknowledge this limitation in our methodology section (and in Table OA3 of the online appendix). When using BRFSS data for county-level analysis, we employ several approaches to address this representativeness concern:

- We include robustness checks where we measure our treatment variable (Republican vote share) at the state level instead of the county level (as shown in Table OA3).
- We use county-fixed effects to control for time-invariant county characteristics that might affect sampling.
- We primarily use BRFSS for our mechanism analysis rather than our main findings, which rely on comprehensive administrative mortality data covering the entire population. We explicitly note this limitation when interpreting results based on BRFSS data.

This representativeness issue is why we triangulate findings using multiple data sources rather than relying solely on BRFSS for our conclusions.

I believe the restricted GSS data do include information on the country of residence. Not that I suggest you should use it but rather mention it if I recall things correctly.

Reply: Thank you for your input. You are correct - the restricted version of the General Social Survey (GSS) includes geographic identifiers, including the county of residence. While we do not use this in our current analysis (especially considering that our treatment in this setting is at the individual level), it is worth noting that the restricted-use GSS data could potentially allow to control for county fixed effects.

The restricted GSS data requires special permission and access through secure data facilities, which is why we opted to use the public GSS data for our supplementary analysis focused on individual-level political preferences rather than geographic variation. However, we appreciate you pointing this out, and we mentioned this possibility in footnote 3 in the revised version of the paper. We will definitely request access to the database if you request us to do so.

Please mention the number of counties in the US in the data section.

Reply: Thank you for this remark. In footnote 4 in the revised version of the paper, we report that our analysis covers a comprehensive set of counties across the United States. More specifically, we report that the United States has 3,143 counties and county equivalents. After accounting for data availability across our various sources and applying our sample selection criteria, our baseline analysis includes 2,416 counties that never changed their political preferences during the 2000-2019 period. This represents approximately 77% of all US counties, providing broad geographic coverage across the nation and ensuring our findings are nationally representative.

Again, we would like to thank you for your feedback, which has helped us to substantially improve our paper.

References

K. Barnatchez, L. D. Crane, and R. A. Decker. An assessment of the national establishment time series (nets) database. 2017.

R. B. Barsky and E. R. Sims. Information, animal spirits, and the meaning of innovations in consumer confidence. *American Economic Review*, 102(4):1343–77, 2012.

B. Callaway, A. Goodman-Bacon, and P. H. Sant'Anna. Difference-in-differences with a continuous treatment. Technical report, National Bureau of Economic Research, 2024.

T. J. Classen and R. A. Dunn. The politics of hope and despair: The effect of presidential election outcomes on suicide rates. *Social Science Quarterly*, 91(3):593–612, 2010.

G. B. Dahl, R. Lu, and W. Mullins. Partisan fertility and presidential elections. *American Economic Review: Insights*, 4(4):473–90, 2022.

L. Goldman, M. P. Lim, Q. Chen, P. Jin, P. Muennig, and A. Vagelos. Independent relationship of changes in death rates with changes in us presidential voting. *Journal of General Internal Medicine*, 34:363–371, 2019.

M. Kivimäki and A. Steptoe. Effects of stress on the development and progression of cardiovascular disease. *Nature Reviews Cardiology*, 15(4):215–229, 2018.

A. Maas and L. Lu. Elections have consequences: partisan politics may be literally killing us. *Applied Health Economics and Health Policy*, 19(1):45–56, 2021.

B. S. McEwen. Protective and damaging effects of stress mediators. *New England Journal of Medicine*, 338(3):171–179, 1998.

M. T. Mefford, M. A. Mittleman, B. H. Li, L. X. Qian, K. Reynolds, H. Zhou, T. N. Harrison, A. C. Geller, S. Sidney, R. P. Sloan, et al. Sociopolitical stress and acute cardiovascular disease hospitalizations around the 2016 presidential election. *Proceedings of the National Academy of Sciences*, 117(43):27054–27058, 2020.

A. Mian, A. Sufi, and N. Khoshkhou. Partisan bias, economic expectations, and household spending. *The Review of Economics and Statistics*, pages 1–46, 2021.

M. Milosh, M. Painter, K. Sonin, D. Van Dijcke, and A. L. Wright. Unmasking partisanship: Polarization undermines public response to collective risk. *Journal of Public Economics*, 204:104538, 2021.

C. Panagopoulos and A. C. Weinschenk. Health and election outcomes: Evidence from the 2020 us presidential election. *Political Research Quarterly*, page 10659129221113256, 2022.

L. Pierce, T. Rogers, and J. A. Snyder. Losing hurts: The happiness impact of partisan electoral loss. *Journal of Experimental Political Science*, 3(1):44–59, 2016.

L. Rosman, E. Salmoirago-Blotcher, R. Mahmood, H. Yang, Q. Li, A. J. Mazzella, J. Lawrence Klein, J. Bumgarner, and A. Gehi. Arrhythmia risk during the 2016 us presidential election: the cost of stressful politics. *Journal of the American Heart Association*, 10(11):e020559, 2021.

C. Templin, J. R. Ghadri, J. Diekmann, L. C. Napp, D. R. Bataiosu, M. Jaguszewski, V. L. Cammann, A. Sarcon, V. Geyer, C. A. Neumann, et al. Clinical features and outcomes of takotsubo (stress) cardiomyopathy. *New England Journal of Medicine*, 373 (10):929–938, 2015.

J. H. Wasfy, C. Stewart III, and V. Bhambhani. County community health associations of net voting shift in the 2016 us presidential election. *PLoS One*, 12(10):e0185051, 2017.
