# Supplementary material for: The health costs of losing political representation: Evidence from U.S. Presidential Elections
Source: PLoS One. 2025 Oct 31;20(10):e0334507. doi: 10.1371/journal.pone.0334507 (PMC12578145; doi:10.1371/journal.pone.0334507)
Supplement: Attachment — Submitted filename: Response to Reviewers.pdf [file pone.0334507.s022.pdf]

Revision of the manuscript PONE-D-24-56123R1

Dear Professor Gutmann,

We want to thank you and the reviewers for your feedback. We have carefully reviewed the manuscript to address the points raised. In the following, we provide a detailed, point-by-point response.

Comment 1: *Please modify the title to ensure that it is meeting PLOS' guidelines (`https://journals.plos.org/plosone/s/submission-guidelines#loc-title`). In particular, the title should be "specific, descriptive, concise, and comprehensible to readers outside the field". In this case, the title is not sufficiently informative and specific about your study's scope and methodology. Adding a descriptive subtitle would be one way to satisfy these requirements.*

Response: Thank you for your guidance. We have changed the title to:

> **"The Health Costs of Losing Political Representation: Evidence from U.S. Presidential Elections"**

This title clearly conveys the scope (health impacts), the key independent variable (loss of political representation), and the empirical context (U.S. Presidential elections). For this reason, we believe it meets PLOS' requirements for specificity, descriptiveness, conciseness, and accessibility to non-specialists.

Comment 2: *You empirical analysis is exploring potential causal mechanisms, but it is missing an explicit statement as to the limitations of this analysis. Some statements in the manuscript might, thus, appear as not being fully supported by the evidence provided. On the one hand, some of these mechanisms, such as social isolation and mental health are not necessarily independent from each other. On the other hand, it cannot be ruled out that important other confounding mechanisms are not accounted for. Different mechanisms are not (even where it would be theoretically plausible) tested against each other. Thus, I*

would ask you to address these limitations, e.g., in the following way:
(i) At the beginning of Section 2.3, clarify that you are highlighting potential mechanisms behind your findings.
(ii) State explicitly in the manuscript that you only show that these potential mechanisms exist, but you are not empirically evaluating their relative importance for mortality by testing them against each other.
(iii) Finally, if you agree that the analysis of mechanisms has these limitations, please tone down the sentence in the conclusion stating that "We further show that political sentiments and social isolation are at the core of our findings." toward something like "these effects appear to be due to ..." or "evidence suggests that ...".

Response: Thank you again for your guidance. We have implemented the requested clarifications:

1. At the start of Section 2.3, we now state: *"The previous section provides evidence that a change in political leadership affects health outcomes. We recognize that multiple factors could simultaneously contribute to this result. Furthermore, our reduced form analysis does not provide us with the capability to disentangle the extent to which each mechanism we investigate can explain the documented results. However, in this section, we still highlight interesting* ***potential*** *underlying mechanisms behind our main finding. "* We also changed our wording throughout the whole paper accordingly.

2. We follow your suggestion, and we added this sentence to the conclusions: *"Unfortunately, we do not empirically assess their relative contribution to the observed effects on mortality, and we cannot exclude the possibility that other mechanisms also play a role."* We further include similar statements in the introduction and throughout the paper.

3. We toned down the original conclusion sentence. It now reads: *"Evidence suggests that political sentiments and social isolation may be important factors underlying our findings."*

We hope that these changes ensure that that our discussion of mechanisms is appropriately framed as exploratory and that our claims are aligned with the evidence presented.

We believe these revisions address the requested changes. We thank you again for your helpful comments and guidance and look forward to the continued evaluation of our manuscript.

Sincerely,

Sris Chatterjee, Iftekhar Hasan, Stefano Manfredonia
